# Faunal input at host plants: Can camel thorn trees use nutrients imported by resident sociable weavers?

**DOI:** 10.1002/ece3.6798

**Published:** 2020-09-21

**Authors:** Kervin D. Prayag, Carla J. du Toit, Michael D. Cramer, Robert L. Thomson

**Affiliations:** ^1^ Department of Biological Sciences University of Cape Town Rondebosch South Africa; ^2^ FitzPatrick Institute of African Ornithology DST‐NRF Centre of Excellence University of Cape Town Rondebosch South Africa

**Keywords:** Arid zone ecology, avian nutrient deposition, ecophysiology, ecosystem engineers, habitat heterogeneity, nutrient cycling, plant–animal interactions

## Abstract

“Islands of fertility” result from the focussing of water and nutrients around many shrub or tree species due to plants foraging for resources. Plant–animal feedbacks may amplify the development of such islands through environmental modification due to, for example, faunal deposition of nutrients and seeds. Fauna residing within vegetation clumps are likely to exert stronger feedbacks on their hosts than itinerant species. We studied the interaction between camel thorn trees (*Vachellia erioloba*) and the colonial nests of sociable weavers (*Philetairus socius*) in the Kalahari. We hypothesized that the accumulation of biological material below the nests will alter the nutrient status of the soil beneath the nest trees, in relation to unoccupied trees and the surrounding grassland. We also suggested that this association will have both positive and negative effects on the camel thorn trees. We found that soil concentrations of N, P, and K were, respectively, 4, 4.6, and 1.2 times higher below trees with nests compared to control trees, indicating faunal concentration of nutrients. Soil δ^15^N values were higher below trees with nests than below control trees without nests. Foliar δ^15^N values were also higher in nest trees than in control trees, showing the trees accessed faunally derived N. Furthermore, foliar biomass per diameter of terminal branches was 27% higher in nest trees, suggesting that trees respond to nutrient input from the weavers with increased growth. Large barren areas in the subcanopy vegetation directly beneath the colonies were attributed to decreased water infiltration rates, as a result of accumulation of organic matter due to continuous deposition of feces, possibly limiting competitive species from establishing in the subcanopy. On the other hand, canopy volume was reduced in trees with nests due to nests occupying large volumes within the canopy, and nests frequently causing branch fall, indicating costs associated with hosting weaver colonies. **Synthesis:** We found nutritional benefits to camel thorn trees when hosting sociable weaver colonies. These benefits can potentially overcome important environmental constraints, but these are partially offset by the resulting costs to the host trees.

## Graphical abstract

The interaction between camel thorn trees and sociable weavers in the Kalahari was investigated. Do the camel thorn trees benefit from the nutrient input by the weavers in return for hosting them in their large canopies? We found that the camel thorns do make use of the faunal nutrient input, but that these benefits do not come without costs.

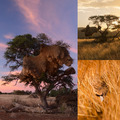

## 1 INTRODUCTION

Ecosystem engineering via highly localized faunal deposition of feces and urine can have dramatic effects on soil properties and vegetation structure and composition (Ellis, 2005; García, Maranon, Ojeda, Clemente, & Redondo, 2002). Seabird colonies change soil nutrient status due to the deposition of guano, leading to increased plant biomass and diversity (Anderson & Polis, 1999; Smith, 1978; Young, 1936; Zółkoś, Meissner, Olszewski, & Remisiewicz, 2013). In contrast, concentrated faunal nutrient input can also lead to a reduction in vegetation cover and species richness (Hobara et al., 2001; Osono, Hobara, Koba, Kameda, & Takeda, 2006). While examples of concentrated faunal input and the impacts on soil nutrient distribution accumulate in the literature (Natusch, Lyons, Brown, & Shine, 2016; Natusch, Mayer, Lyons, & Shine, 2017; Pinkalski, Damgaard, Jensen, Peng, & Offenberg, 2015; Sekercioglu, Wenny, & Whelan, 2016; Smith, 1978), we still know very little about the consequences for the host vegetation. Furthermore, little is known about the impacts of faunal concentration of nutrients on soil nutrient distribution in the broader landscape, especially in arid environments where outcomes are predicted to differ from more benign environments (i.e., “stress gradient hypothesis,” Bertness & Callaway, 1994).

The net outcome of interactions between plants and animals is not obvious. Often both facilitative and negative effects are evident, perhaps at different temporal or spatial scales, meaning that the ultimate fitness consequences for individuals may change depending on timing or conditions (Bronstein, 2015; Grinath, Larios, Prugh, Brashares, & Suding, 2019; Hernändez, Sanders, Miller, Ravenscraft, & Frederickson, 2017). Despite the prevalence of facilitative interactions between plants and animals in arid ecosystems (Boeken, Shachak, Gutterman, & Brand, 1995; Pellmyr, 2002; Rohner & Ward, 1999; Whitney, 2002), there are also multiple examples of negative interactions. For example, herbivory commonly negatively affects plants (Belsky, 2002; Ferraro & Oesterheld, 2002), although fauna can also suffer negative consequences due to encroachment of woody vegetation in response to grazing pressure (Archer et al., 2017; O’Connor, Puttick, & Hoffman, 2014; Venter, Cramer, & Hawkins, 2018). Gaining an understanding of the interplay and outcomes of interactions between species in specific systems and contexts is important to recognize their broader effects in the ecosystem.

Trees hosting colonies of animals for long periods of time is a frequent occurence in nature, and are interesting direct plant‐animal interactions. These interactions are however rarely studied in detail, especially from the trees' perspective. In the arid and semiarid regions of Southern Africa, the camel thorn (*Vachellia erioloba)* is an iconic and dominant savanna tree species growing up to 12 m tall (Coates Palgrave, 2005). In these regions, camel thorns engineer the ecosystem by creating “islands of fertility” (sensu Schlesinger et al., 1990), thus fostering the growth of other smaller plant species below their large canopies (Dean, Milton, & Jeltsch, 1999). camel thorns also provide habitat and shelter for many different animal species, including birds and mammals looking for shade to escape the heat of the day (Dean et al., 1999). For this reason, camel thorns are thought to be critical keystone species for animal and plant communities in these regions.

**Figure 1:**
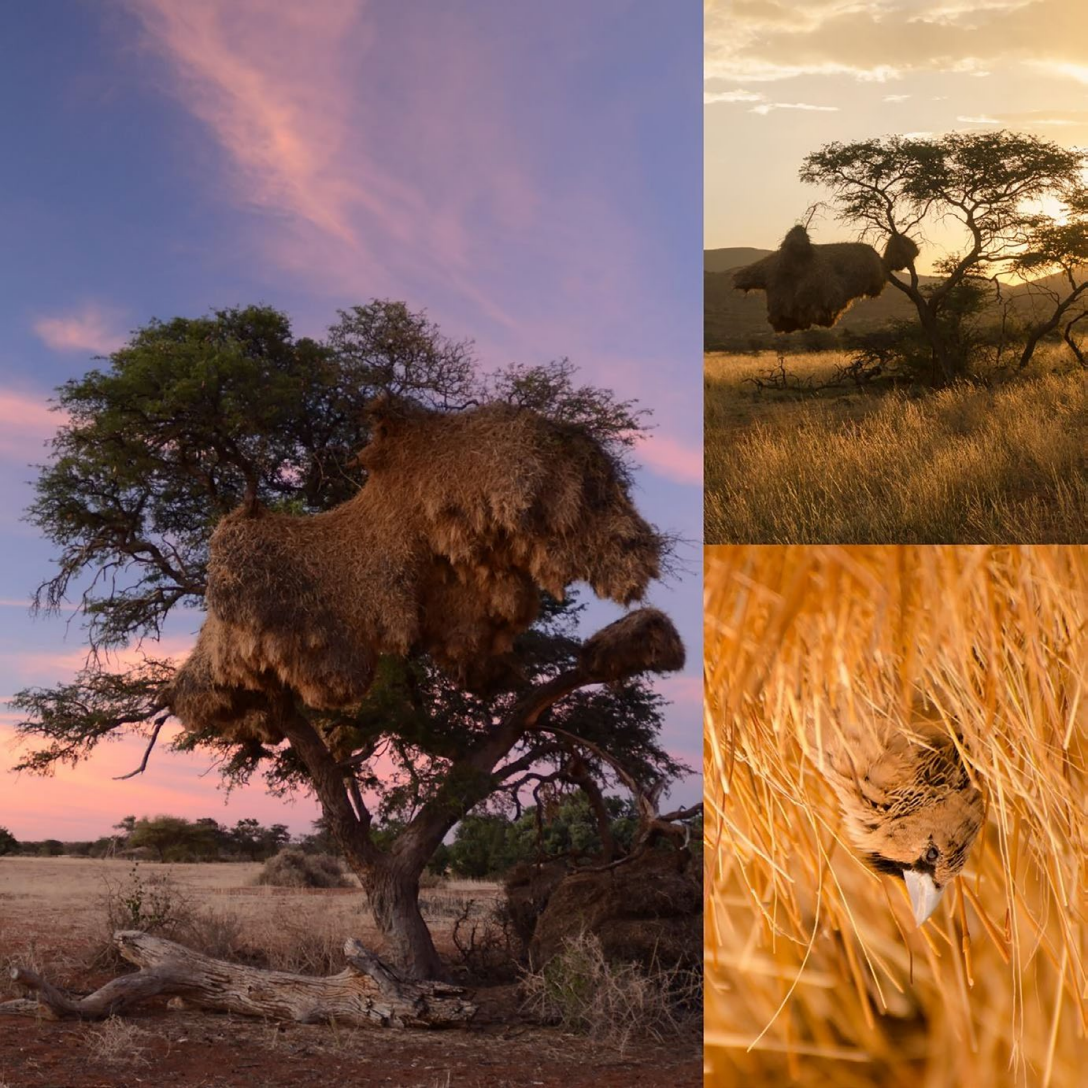
Left and top right panes: sociable weaver colony nests on camel thorn trees at Tswalu Kalahari. Right bottom pane: sociable weaver coming out from nest chamber at Tswalu Kalahari. Photo credits: Anthony M. Lowney

Large communal nests of sociable weavers (*Philetairus socius*) often occur in camel thorns (see Figure 1; Dean et al., 1999; Seymour, 2008). These small passerines build massive colonial nests, where they live throughout the year in groups of up to 300 birds (Mills & Mills, 2013). The colonies can persist for several decades (Maclean, 1973), and the continuously falling feces, carcasses, and nest material can potentially have important consequences on the soil properties and vegetation below and around the trees (Dean et al., 1999). Soil properties that may be impacted include texture, with the large weaver nests trapping and concentrating finer sand or soil particles below the canopy of host trees, as also happens in other vegetation clumps (Cramer & Midgley, 2015). The localized and continuous deposition of feces and nest material over long periods may increase soil organic matter which can lead to reduced water infiltration, as also occurs in other systems (Shakesby, Doerr, & Walsh, 2000). This faunal input can also increase the soil concentrations of a range of nutrients (Anderson & Polis, 1999; Smith, 1978; Zółkoś et al., 2013). Camel thorn trees may be able to acquire and make use of these faunal‐derived nutrients from below their canopies, potentially alleviating nutrient acquisition constraints (Dean et al., 1999).

There are also potential negative effects of the sociable weaver interaction for camel thorns. Sociable weaver nests are highly flammable, resulting in complete destruction of trees during occasional fires (Seymour & Huyser, 2008). The nests are also continuously expanded by the weavers over time, becoming massive with age (Maclean, 1973), and it is common to observe broken branches associated with larger nests. Finally, the nests occupy space in the canopy, blanketing branches with nest material that might eliminate potentially productive leaf material. These putative negative interactions between camel thorn trees and sociable weaver nests seem one‐sided, in that the trees bear the costs of accommodating the colonies, but it is hard to imagine the cost to the colonies of potential facilitation of the tree. While there has been consideration of the facilitative interactions between camel thorn trees and sociable weavers (Dean et al., 1999), there has been no consideration of the potential costs versus benefits of this facilitation for the tree.

**Figure 2:**
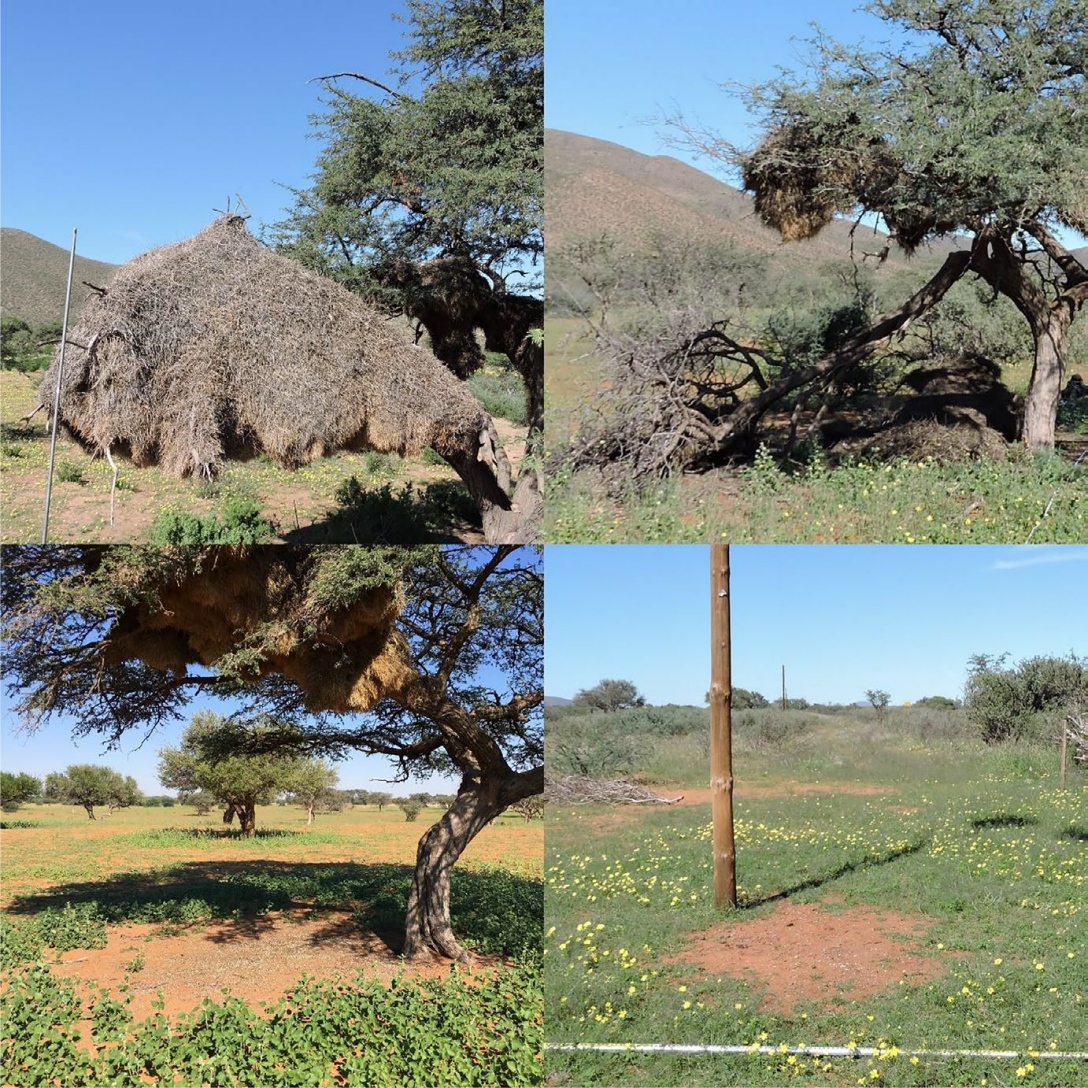
Top left pane: complete replacement of foliage on a branch of a camel thorn tree due to the presence of a sociable weaver nest. Top right pane: branch breakage in a camel thorn tree as a direct consequence of hosting a sociable weaver nest. Bottom left pane: barren area in the vegetation directly below a sociable weaver nest colony below a camel thorn tree. Bottom right pane: barren area in the vegetation directly below a sociable weaver nest colony below a telephone pole

We hypothesized that sociable weaver colonies provide nutrient inputs below camel thorn trees, which benefits the growth and nutritional status of the host trees. We also hypothesized that these positive growth‐related effects may be offset by negative consequences of housing the nests through branch breakage and reduced photosynthetic area by blanketing significant proportions of the tree canopy (Figure 2). To test these hypotheses, soil properties, foliar nutrient status, and foliar canopy traits were compared between host and control trees.

## 2 Methods

### 2.1 Study system

The study was undertaken in Tswalu Kalahari Reserve in the Northern Cape, South Africa (27°13′30″S and 22°28′40″E). This semiarid savanna receives a mean annual precipitation of 361.44 mm ± 169.2 mm (South African Weather Service, 2020). It is characterized by reddish‐brown sandy soils (Davis, Scholtz, Kryger, Deschodt, & Strümpher, 2010) with an open canopy of trees and shrubs that commonly includes camel thorns, blackthorns (*Senegalia mellifera),* and shepherd's trees (*Boscia albitrunca*), surrounded by a sparse grassy layer (Mucina & Rutherford, 2006).

### 2.2 Study design

We selected 18 camel thorn trees containing sociable weaver nests in the study area. We limited our choices to an area of the study site with sandy soils away from rocky mountain outcrops and sand dunes, to keep the environmental conditions as homogeneous as possible. In addition, we selected trees with weaver nests with more than 30 chambers (mean ± SE = 71 ± 28 chambers per nest; range: 33–125 chambers per nest) to ensure adequate faunal nutrient input. Each nest‐containing tree was paired with a nearby control tree without a nest. The paired camel thorn trees with and without nests were no more than 200 m apart and had similar heights and trunk diameters at breast height (*p* > .426 and *p* > .253, respectively). The main branch on which the nest was built was identified and matched with a similar‐sized and oriented branch on the control tree. We calculated cross‐sectional areas of the main tree trunks and of the main branch using diameter at breast height and the basal diameter of the nest branch, respectively. The heights of all 36 trees were measured using a hypsometer (Nikon Forestry Pro, Nikon Vision Co., Ltd, Tokyo, Japan).

Five sociable weaver nests on telephone poles were also included in the study. Only five pole nests were sampled as suitable poles are rare in the reserve and we wanted to restrict our sampling to a narrow edaphic and climatic region. In each case, the nest poles were paired with a neighboring control pole without a nest. These pole nests were included to control for the influence of the camel thorn trees, per se, on soil properties. It has been shown that mammalian activity increases around a camel thorn tree hosting a nest (e.g., for shading to avoid heat stress, Dean et al., 1999; Lowney, 2020). As such, the telephone poles also served as a means of isolating the effects of the sociable weaver feces on soil chemistry from the effects of mammalian input. Directly beneath each weaver nest, whether on a camel thorn tree or a telephone pole, we observed the presence of an area barren of any vegetation (Figure 2).

### 2.3 Soil sampling procedures

For each tree pair, we collected soil samples from five specific locations. These were (a) in the barren area directly below the nest, (b) in the corresponding site under the equivalent branch of the control tree, (c) just outside the barren areas directly below the nests, (d) at the same distance from the base of the control tree trunk, and (e) in the open grassland located halfway between the control and nest trees. At each of the five locations, soil was collected at three depths: the top 20 cm, 30–50 cm, and 100–120 cm deep, using a bucket auger (20 long × 5 cm diameter). Soil samples were collected at these same depths below the telephone pole pairs from two specific locations. These were (a) in the barren area directly below the nest and (b) under the paired control pole without a nest. Multiple auger volumes of surface soil (i.e., top 20 cm) were also collected at each nest and control tree site for plant growth experiments (ca. 2.5 kg, see below).

All the soil samples were air‐dried in the open after which they were sieved through a 2‐mm mesh. A quartering method was then used to divide up the soil samples for the various tests (Gerlach, Dobb, Raab, & Nocerino, 2002). Air‐dried soils were submitted for nutrient analyses and mass‐spectrometer analysis of isotopes. Soil samples from the 10 tree pairs, which included the nests having the highest number of chambers, were selected for soil physical and chemical analyses (see below). All five soil samples from below all the telephone poles were analyzed. Subsamples from these selected soil samples were combusted at 400°C in a furnace for 24 hr to remove organic carbon prior to particle size and elemental analysis (see below).

### 2.4 Soil particle size distribution

Soil particle size was measured from combusted soil samples for the top 0.2 m of the soil using a Malvern Mastersizer 2000 (Malvern Instruments Ltd, Malvern, UK). Each sample was measured thrice, and the average taken. The proportions of the soil samples falling into each size class were measured and recorded, and the soil was further classified into seven categories representing silt, clay, and sand according to the Wentworth grain size chart (Williams et al., 2006).

### 2.5 Fecal sampling procedure

Rectangular plastic trays (30 × 50 cm) were placed directly beneath 10 nests in camel thorn trees just before sunset to collect fresh sociable weaver feces. Feces were collected from the trays the next morning, and immediately placed in a freezer to prevent further decomposition. The fecal samples were then freeze‐dried prior to submitted for nutrient analyses and mass‐spectrometer analysis of isotopes (see below).

### 2.6 Water infiltration rates

The water infiltration rate into the soils in the barren areas below the nest, below the control trees and in the grassland was measured using a Mini Disk Infiltrometer (disk radius 2.25 cm; Decagon Devices Inc., Pullman, WA, USA), with a suction head set to 2 cm. Under nest trees, the infiltrometer was placed on top of the fecal mat (absent under control trees and grassland areas). The infiltrometer was filled to 90 ml and the standard operating procedures were used, as detailed in the manual (Decagon Devices, 2016). Readings were taken every 10 s for 15 mins, or until the infiltrometer was empty, whichever came first. To calculate the infiltration rate (K) for each sample, the Excel spreadsheet provided by the manufacturer was used with the parameters for sand (i.e., *a* = 0.145 and A = 2.68).

### 2.7 Phytometer experiment

To test the capacity of the soil to support plant growth, we set up plant growth experiments using wheat (*Triticum aestivum*, cv SST015) as a phytometer for the growth of annuals. Seeds were germinated in vermiculite in a greenhouse, and then transferred to a growth chamber set at 25°C, with 12/12 hr light‐dark cycles with photosynthetically active radiation of *ca*. 250 µmol m^−2^ s^−1^. Once the seedlings had grown to a height of ca. 10 cm, they were transplanted in 15‐cm‐diameter pots, respectively, filled with soils taken 1—from the barren areas below nest trees, 2—from below control trees, 3—from the surrounding grassland, 4—from below nest poles, and 5—from below control poles. Three seedlings of approximately the same size were planted in each pot, 10 cm apart from each other and at a depth of 3 cm. The pots were supplied 200 ml of water, and the heights of the plants were measured every second day. After 8 days, the aboveground matter of one plant from each pot was harvested, weighed, oven‐dried at 70°C for 48 hr, and reweighed to allow for the calculation of water content and dry mass. This aboveground harvest and the subsequent measurements were repeated after 2 weeks on the second plant from each pot. The third plant in each pot was harvested from the soil after three weeks.

### 2.8 Foliar sampling procedures

We sampled the terminal branches from each of the four cardinal directions from the canopy of each tree (18 nest trees and 18 control trees). We stripped these branches of their leaves and measured the fresh weight of the leaves. We also measured the subtending branch diameter and length. The fresh weight of the leaves was expressed as a function of the length and basal diameters of the respective terminal branches. The leaves originating from each tree were then pooled, and we dried these in a domestic oven at ca. 70°C for 36 hr. The dry weights of the leaves were recorded, prior to being ground to a fine powder using a ball mill. The powdered leaf samples were submitted for nutrient analyses and mass‐spectrometer analysis of isotopes (see below).

Using a digital camera, lateral view photographs of the nest branches (and their control tree branch equivalents) were taken with a 1‐m rule in the frame for scale. A grid of 10 × 10 cm cells was then overlaid onto the photographs using Adobe Photoshop CS5 Extended (Adobe Systems Incorporated, Version 12.0) and each grid cell coded according to their content (i.e., >50% nest or leaf). The coded cells were then counted using the “Magic Wand” and “Count” tools. From these counts, nest and foliar two‐dimensional areas on nest branches were obtained. These two‐dimensional areas were standardized by the cross‐sectional areas at the base of the subtending branches.

For each tree, the number of fallen branches was recorded, and the branch basal diameters were measured. The summed cross‐sectional area of all fallen branches for each tree was calculated and standardized against the respective cross‐sectional areas of the main trunk of the tree from which they had fallen.

### 2.9 Elemental, nutrient, and isotopic analyses

The combusted soil samples, frozen fecal samples, and dried foliar samples were ground to a fine powder using a mortar and pestle or a ball mill (MM200, Retsch, Germany). The powdered samples were placed in Perspex rings sealed with 4‐μm Polypropylene Thin Film (Chemplex Industries Inc, Florida, USA) and introduced to a SPECTRO XEPOS XRF spectrometer (SPECTRO, AMETEK materials analysis division, Kleve, Germany). Analyses were conducted using the X‐LabPro 5 software, which incorporates the universal “Turbo Quant Powders” method. The instrument was calibrated by using a certified standard GBW07312 (National Research Center for CRMs, Beijing, China), for which elemental concentrations were obtained from NOAA Technical memorandum NOS ORCA 68 (1992). Only the elements that were within the machine's detection limits were included.

Uncombusted sieved surface soil samples (to depth of 0.2 m) in the barren patches and corresponding adjacent areas were submitted to the Elsenburg Laboratory (Western Cape Department of Agriculture, Stellenbosch, South Africa) for further nutritional analyses of plant‐available nutrients. Analyses included pH, electrical conductivity, and the concentrations of Mg, Na, K, citric acid‐extractable P (1% (w/v) citric acid), and Olsen P following protocols of soil science society of South Africa (1990). The pH was measured in 1 M KCl extracts. Electrical conductivity was measured on a soil paste, obtained by mixing the soil samples with deionized water.

The δ^15^N values and the total N concentration of the uncombusted sieved soil, dried foliar, and fecal samples were determined using mass spectrometry. Samples were weighed into tin capsules (5 × 9 mm; Säntis Analytical, Teufen, Switzerland, with ca. 10 μg of soil powder, 1 μg of fecal powder, and 2 mg of foliar powder used for analysis). The samples were combusted in a Flash 2000 organic elemental analyzer, and the gases passed into a DELTA V Plus isotope ratio mass spectrometer (IRMS) via a ConFlo IV gas control unit (all from Thermo Scientific, Bremen, Germany). In‐house standards and one IAEA (International Atomic Energy Agency) standard (USGS25) were used to calibrate the results. Nitrogen concentration was expressed relative to atmospheric nitrogen (Evans, 2001).

### 2.10 Data analyses

All statistical analyses were performed using the R statistical software (R Core Team, 2020).

Comparisons were made using linear mixed‐effect models (LMMs) fitted by restricted (residual) maximum‐likelihood estimation (REML). The LMMs were run in R using the package “*lme4’’* (Bates, Mächler, Bolker, & Walker, 2015).

For each tree pair, the physical and chemical properties of the top soil layer sampled at the different locations were compared. Random effects were included to account for the nonindependent structure of our sampling design wherein samples in a triplicate or pair (i.e., nest trees, control trees, and surrounding grassland, or control and nest poles) are likely more similar to each other than to trees or poles in other triplicates or pairs due to environmental heterogeneity. The same model structure was used to compare the chemical properties of the top soil layer below nest and control telephone poles. Separate comparisons of trees and telephones poles were done because poles were located spatially away from the rest of the study area where climatic and edaphic properties may be different.

Variations in soil chemical properties with depth were compared between nest trees, control trees, nest poles, control poles, and the surrounding grassland with triplicates/pairs included as random effects. Variations in aboveground biomass of the wheat plants from the phytometer experiments were analyzed, with time of harvest and triplicates/pairs included as crossed random effects. Water infiltration rates into the soil were compared between the barren areas below nest trees, below control trees, and in the grassland, with triplicates included as random effects. Foliar physical and chemical properties were compared between control trees and nest trees.

Normality of data was visually assessed prior to fitting the models. Data were log‐transformed where appropriate to fit the constraints of the models. Diagnostic plots of the models were generated to check for nonlinearity, unequal error variances, and outliers. In instances where random effects were too complex to be supported by the data, models with singular fits were generated (i.e., without random effects). Type III analyses of variance (with Satterthwaite's method) were done on all the fitted models to determine the significance of the main effects using the “*car”* package (Fox & Weisberg, 2019). Where main effects significantly explained variation, pairwise comparisons (Tukey's post hoc tests) of group levels were then performed using the package “*emmeans”* in R (Lenth, 2019). Confidence interval (95%) ribbons on plots were calculated using nonparametric bootstrapping with 1,000 replicates using the “*Hmisc”* package in R (Harrell & Dupont, 2019). When comparing sample means, if 95% confidence intervals (CI) overlap by no more than half then *p* ≤ .05, and if 95% CIs do not overlap, then *p* ≤ .001 (Cumming & Finch, 2005). All plots were generated using the “*ggplot2”* package in R (Wickham, 2016).

## 3 RESULTS

### 3.1 Soil characteristics

**Figure 3:**
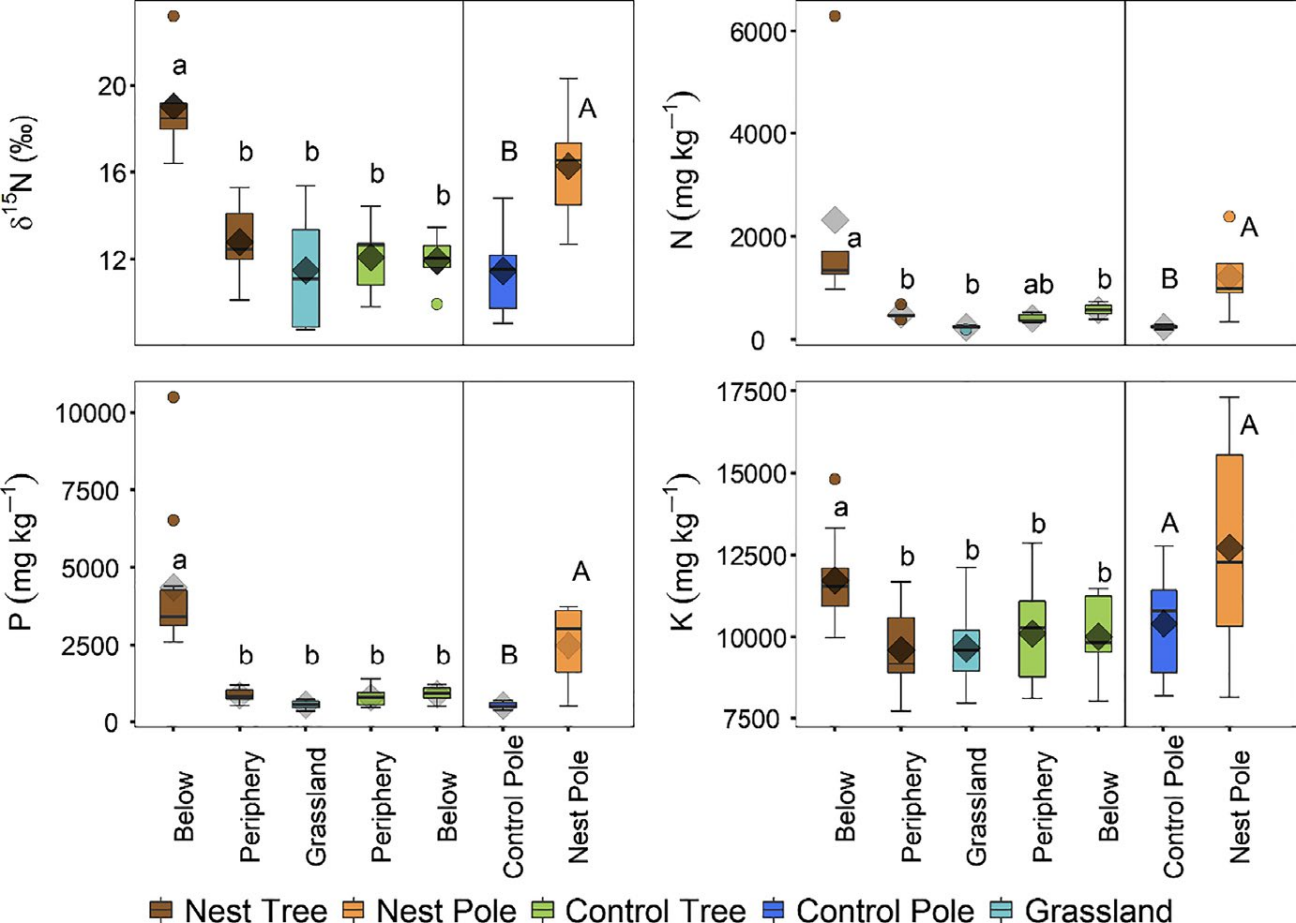
Variation in total N, P, and K concentrations, and in δ^15^N in the top soil layer (0.1 m) in the barren areas directly below the nests, below control trees, in the periphery of both nest and control trees, in the grassland, and below nest and control telephone poles. The boxes and horizontal lines, respectively, represent the first and third quartiles, and the medians. The whisker represents 1.5 × the interquartile range, and outliers above/below are shown as open circles. The diamonds represent the mean values. Different letters indicate significant differences as determined by Tukey's pairwise comparisons

Below nest trees, the total concentrations of N, P, K, Ca, Cu, and Zn in the top soil layer were found to be significantly higher in the barren areas directly below the nests than in the peripheral area surrounding them, the control trees and the grassland (Figure 3; Appendix S1: Fig. S1 & Table S1). The soil δ^15^N values directly below the nests were also significantly higher than in the periphery, control trees and the grassland (Figure 3; Appendix 1: Table S1). For the control trees, there was no significant difference in total nutrient concentrations or δ^15^N between the area directly below the tree and the peripheral area. Furthermore, N, P, K, Ca, Cu, and Zn, as well as δ^15^N, were not significantly different between control tree sites and the surrounding grassland. The top soil layer below telephone poles with nests also had higher δ^15^N, N, P, Ca, Cu, and Zn than did the top soil layer below neighboring poles without nests, but no significant difference was found in K concentration (Figure 3; Appendix S1: Figure S1 and Table S2).

**Figure 4:**
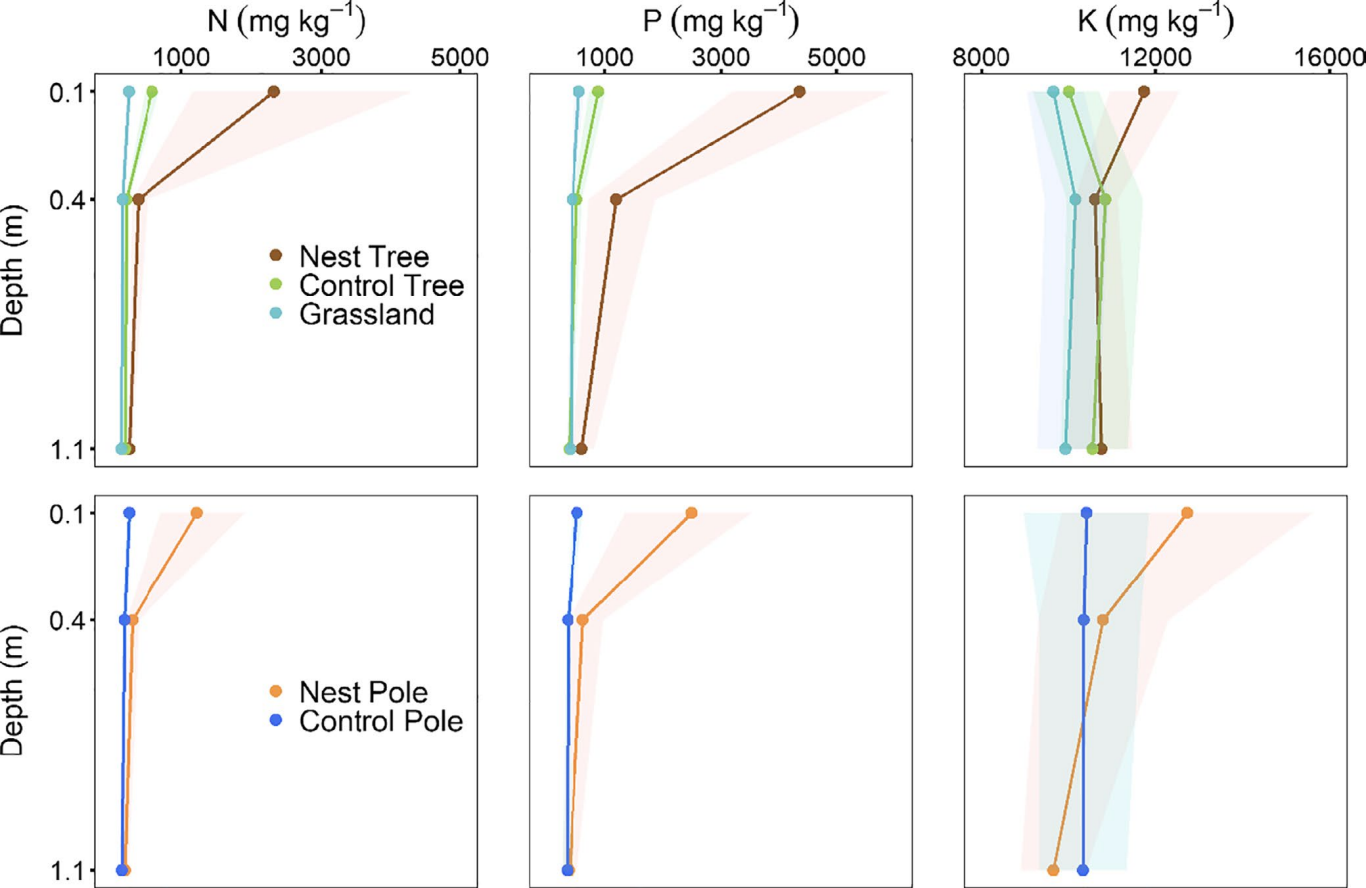
Soil concentrations of total N, P, and K at depths of 0.1, 0.4, and 1.1 m below camel thorn trees (with and without sociable weaver nests) and in the open grassland, and below telephone poles, with and without nests. Points represent means, while colored ribbons represent the bootstrapped 95% confidence intervals

**Figure 5:**
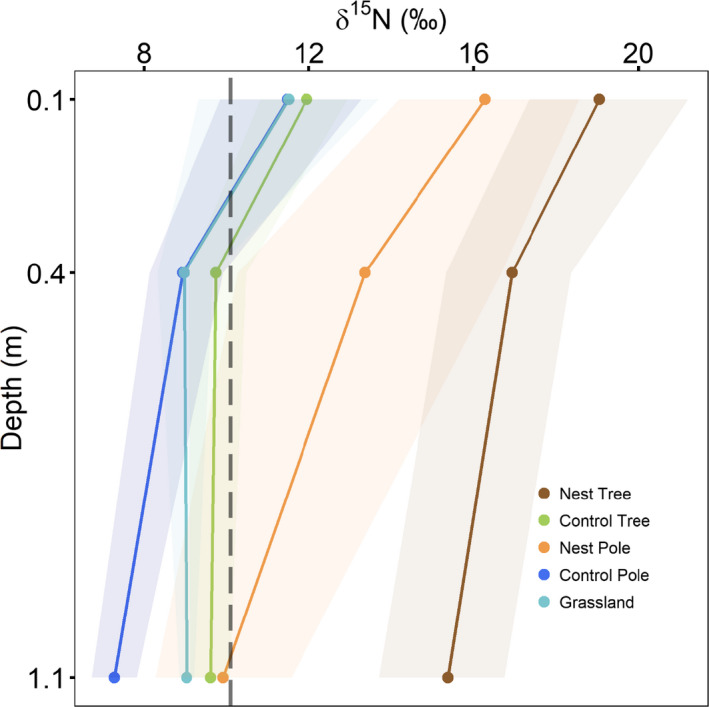
The variation in soil δ^15^N values at depths of 0.1, 0.4, and 1.1 m, between soils under camel thorn trees (with and without sociable weaver nests), telephone poles (with and without nests), and in the open grassland. The vertical dashed line represents the mean δ^15^N value of the sociable weaver fecal samples (10.10‰ ± 0.35). Points represent means, while colored ribbons represent the bootstrapped 95% confidence intervals

Overall, the distribution of soil nutrients varied little with depth and in the absence of nests (Figure 4; Appendix S1: Figures S2 & S3 and Tables S3 & S4). The effects of nests on total nutrient concentrations were restricted to the top soil layer, as no differences were observed between nests and controls (trees or poles) at deeper soil layers (i.e., between 0.4 and 1.1 m below the surface). Soil δ^15^N values were, however, increased throughout all the depths sampled below trees with nests, and up to 0.4 m below poles with nests (Figure 5; Appendix S1: Tables S3, S4 & S7). δ^15^N values were statistically indistinguishable between the control trees, the grassland sites, and the control poles (Figure 5; Appendix S1: Tables S3, S4 & S7).

**Figure 6:**
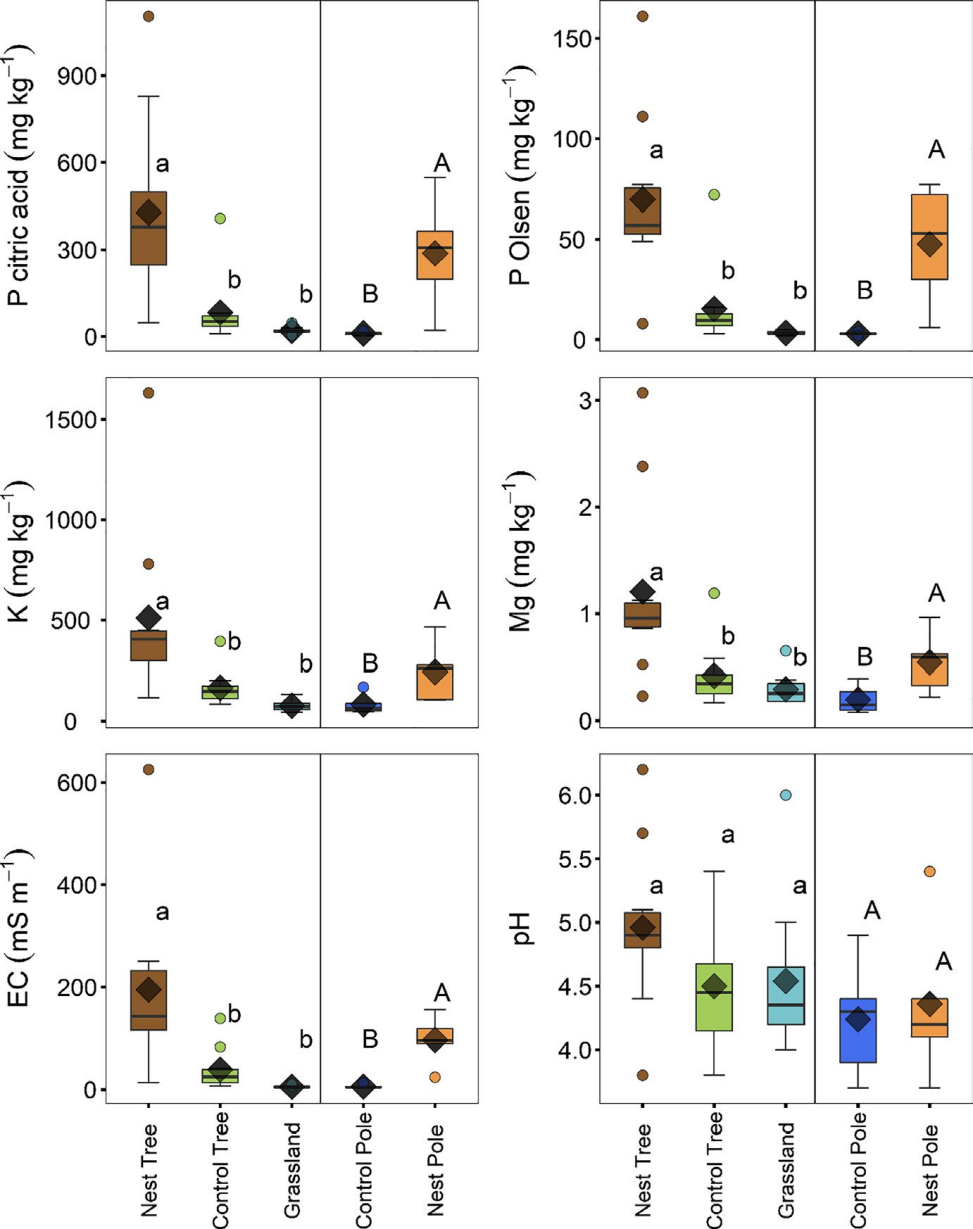
Soil concentrations of P (citric acid extraction), P‐Olsen, K, and pH, and electrical conductivity in the top soil layer (0.1 m) below camel thorn trees with and without sociable weaver nests, in the grassland, and below telephone poles with and without nests. The boxes and horizontal lines represent the first and the third quartiles and the medians, respectively. The whisker represents 1.5 × the interquartile range and outliers above/below are shown as open circles. The diamonds represent the mean values. Different letters indicate significant differences as determined by Tukey's pairwise comparisons

Plant‐available nutrients were measured to determine the availability of nutrients for plant growth. In the top soil layer, higher concentrations of citric P, Olsen P, extractable K and Mg, and increased electrical conductivity (EC) were measured below trees with nests compared to control trees and the grassland (Figure 6; Appendix S1: Table S5). Similarly, concentrations of these nutrients and electrical conductivity were higher below poles with nests relative to control poles (Figure 6; Appendix S1: Table S6). No differences in the concentrations of any of the nutrients or soil conductance were found between the soils below the control trees and the grassland soil, and no difference was found in soil pH at any of the locations (Figure 6; Appendix S1: Table S5 & S6).

### 3.2 Soil particle size distribution

The soil samples were categorized as sandy soils, with the main component of the soil being classified as fine sand (Appendix S1: Figure S4; Table S10). Mineral particle composition of the coarser fractions of soil did not differ between the grassland, below control trees or nest trees. However, there were significantly higher concentrations of the finer particles (i.e., clay and silt) in the soil below the control trees and nest trees than in the grassland and also below poles with nests relative to poles without nests.

### 3.3 Fecal matter

All the nutrients that were enriched in the top 0.1 m of soil below nest trees and nest poles were also found in high concentrations in the sociable weaver feces (Table 1). The δ^15^N value for the collected fresh fecal matter was 9.99 ± 1.00‰ (Table 1). This value is lower than that observed for the soil below the trees and poles.

**Table 1:** The mean δ^15^N values (‰) and nutrient concentrations (%) of sociable weaver feces, with standard errors (*n* = 5)

	Fecal value/concentration
δ^15^N	9.99 ± 1.00
*N*	4.89 ± 0.55
P	0.87 ± 0.21
K	1.11 ± 0.29
Mg	0.34 ± 0.14
Ca	0.81 ± 0.37
Cu	0.005 ± 0.003
Zn	0.015 ± 0.002

### 3.4 Infiltration rates

**Figure 7:**
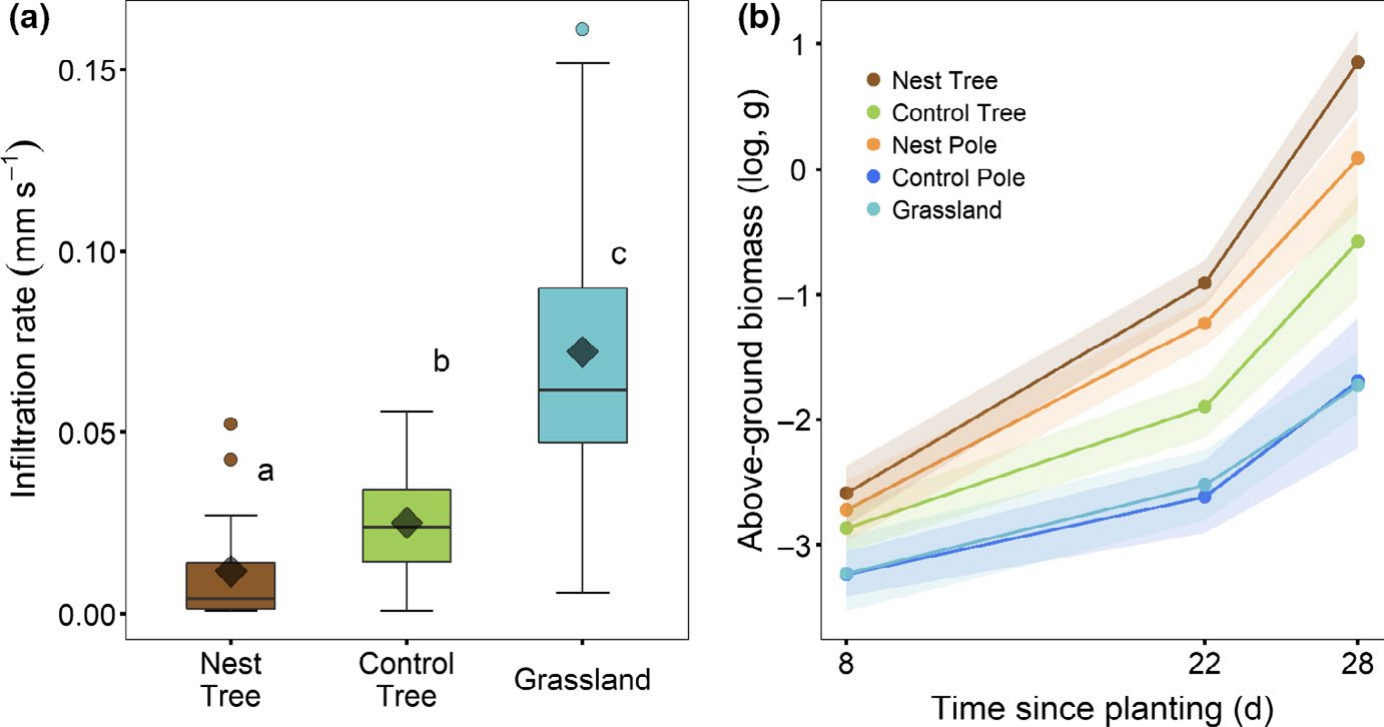
(a) Infiltration rate in the top soil layer (0.1 m) below camel thorn trees with and without sociable weaver nests, and in the open grassland. The boxes and horizontal lines represent the first and the third quartiles and the medians, respectively. The whisker represents 1.5 × the interquartile range, and outliers above/below are shown as open circles. The diamonds represent the mean values. Different letters indicate significant differences as determined by Tukey's pairwise comparisons and (b) mean cumulative change (since planting) in aboveground biomass (logged) of wheat grown in soils collected below camel thorn trees with and without sociable weaver nests, telephone poles with and without nests, and from the open grassland. Points represent means, while colored ribbons represent the bootstrapped 95% confidence intervals

Water infiltration rates into the surface soil in the grassland, below control trees, and in the barren areas directly beneath the colonies were found to be significantly different from each other (Figure 7a; Appendix S1: Table S7). Surface soil infiltration rates in the grassland were found to be the highest, while the lowest rates were recorded in the barren areas (Figure 7a; Appendix S1: Table S7).

### 3.5 Phytometer growth

Differences in wheat biomass accumulation increased steadily from the first harvest after 7 days through to the final harvest after 22 days (Figure 7b; Appendix S1: Table S7). Wheat plants grown for 22 days in soils from directly below nests (both in trees and on telephone poles) accumulated significantly higher aboveground biomass than those grown in soils from below control trees, from below control poles, and from the grassland (Figure 7b; Appendix S1: Table S7). After 22 days, wheat grown in soil from below control trees also had higher biomass than those grown in soil from the grassland (Figure 7b; Appendix S1: Table S7).

### 3.6 Tree characteristics

**Figure 8:**
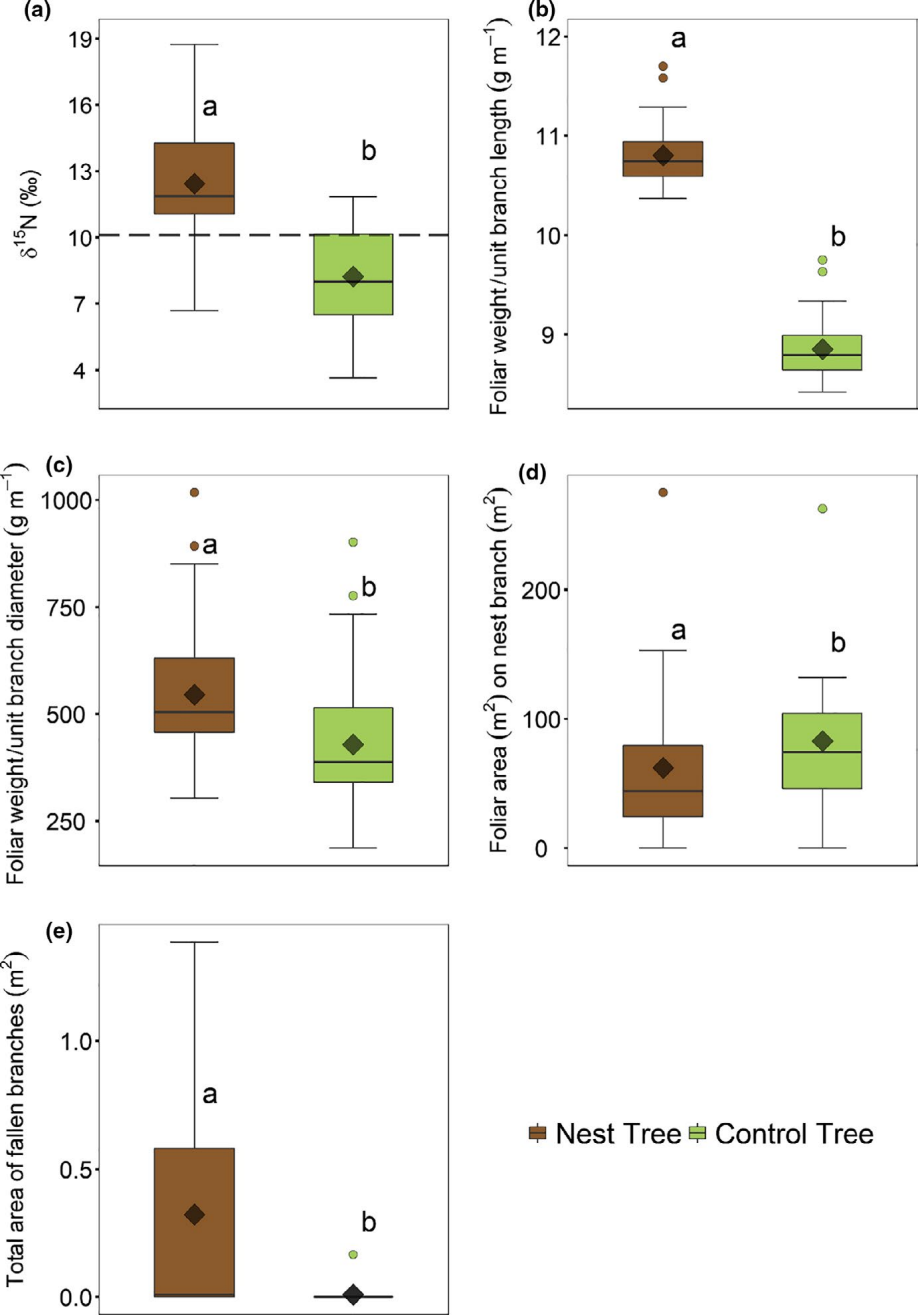
(a) The variation in foliar δ^15^N between camel thorns with and without nests. The horizontal dashed line represents the mean δ^15^N value of the fecal samples, (b) predicted foliar dry weight expressed as a function of the respective terminal branch lengths for camel thorn trees with and without sociable weaver nests as predicted by the fitted linear mixed‐effect model, (c) foliar dry weight expressed as a function of the respective terminal branch basal diameters as predicted by the fitted linear mixed‐effect model, (d) two‐dimensional foliar areas expressed as a function of the cross‐sectional areas of the respective “nest branches” on camel thorn trees with and without nests, and (e) cross‐sectional area of fallen branches expressed as a function of cross‐sectional areas at breast height of respective camel thorn trees with and without nests. The boxes and horizontal lines represent the first and the third quartiles and the medians, respectively. The whisker represents 1.5 × the interquartile range and outliers above/below are shown as open circles. The diamonds represent the mean values. Different letters indicate significant differences as determined by Tukey's pairwise comparisons

Foliar δ^15^N was higher in nest trees compared to control trees (Figure 8a; Appendix S1: Table S8). In contrast, there were no differences in mean foliar concentrations of N, P, K, Mg, Ca, Cu, Zn, or Fe between trees with and without nests. The exception was Mn, which was higher in the leaves of nest trees (Appendix 1: Figure S5 & Table S8). Mean foliar dry weight per unit length of branch and foliar dry weight per unit diameter were higher in trees with nests compared to trees without nests (Figure 8b, c; Appendix S1: Table S9). The branches supporting nests, however, showed a significant decrease in foliar area compared to branches of similar diameter on the control trees (Figure 8d; Appendix S1: Table S9). The summed cross‐sectional areas of fallen branches of nest trees were significantly higher than that of the control trees (Figure 8e; Appendix S1: Table S9).

## 4 DISCUSSION

We found evidence that nutrients deposited as feces by sociable weavers below their nests are incorporated into camel thorn trees. Foliar δ^15^N values approaching zero are generally taken as an indicator of N_2_ fixation in *Vachellia spp*. (Cramer, Chimphango, De Fortier, Waldram, & Bond, 2007). Although camel thorn trees can fix N_2_ (Barnes, Fagg, & Milton, 1997; Burke, 2006), this process can be costly (e.g., ca. 25% of daily photosynthate, Lambers, Chapin, & Pons, 2008) and may be inhibited in instances where availability of mineral N is high (Aranibar et al., 2003). The fact that the δ^15^N of nest trees was significantly greater than that of control trees may thus indicate that the nest trees were less dependent on N_2_ fixation than the control trees. Additionally, the δ^15^N differences may be driven by the consumption of highly fractionated N (i.e., high δ^15^N values, Figure 3) from sociable weaver (Table 1) and other feces. Furthermore, the 22% and 27% increase in dry foliar biomass, respectively, per unit length and per unit diameter of terminal branches on host trees relative to control trees, provide additional evidence of the usage of nutrients input by sociable weavers. Despite this increase in foliar biomass, foliar nutrient concentrations in camel thorn trees did not differ between those with nests and those without. This is likely because the increased growth resulted in “dilution” of the additionally available nutrients (Lawrence, Cooke, Greenwood, Korhnak, & Davis, 2001; Millard, 1988; Tripler, Canham, Inouye, & Schnurr, 2002).

Soil N, P, and K concentrations under the nests were, respectively, 4, 4.6, and 1.2 times higher than under control trees, and 12, 7, and 1.2 times higher than in the grassland surface soils. The enrichment of nutrients in soils directly below nest trees hosting a sociable weaver colony is most likely due to faunal nutrient input. It is not easy to untangle whether the quantitative contributions of avian or mammalian inputs contribute most to this enrichment. However, the high concentrations of the same nutrients in the soil as in the birds' excretions suggest that the continuous deposition of feces by the sociable weavers may contribute significantly to this enrichment. The similar nutrient enrichment pattern below telephone poles with nests versus those without also suggests that sociable weavers greatly increase nutrient concentrations in the soil below their colonies, even in the absence of mammalian activity. This indicates that the weaver feces act as a fertilizer for an entire suite of nutrients for plant growth. This localized input of nutrients can have important implications in arid environments, particularly in terms of N, which acts as the main limiting factor for many plants in the Kalahari (Tilman, Wedin, & Knops, 1996).

Higher δ^15^N values in the soil below the nests compared to the control soils provide further evidence that the sources of N in these soils are different. These results are consistent with other studies, which have shown that δ^15^N values of the soil and plants growing adjacent to large colonies of seabirds differ from the values of those in areas away from the colonies (Erskine et al., 1998; Mizutani, Hasegawa, & Wada, 1986). However, our results show that the δ^15^N values of the enriched soils were higher than in the sociable weaver feces. The reason for this difference is most likely due to the main N component of bird feces being urea (Erskine et al., 1998), which once broken down forms NH_3_. Loss of the lighter isotopes of NH_3_ will occur rapidly (Schlesinger et al., 1990), resulting in fractionation enriching the ^15^N in the soil (Kirshenbaum, Smith, Crowell, Graff, & McKee, 1947) and causing high δ^15^N values in the foliar biomass of trees with nests. Overall, the isotope data indicate significant contributions of nests, whether on poles or trees, to the N in soil and consequently to the trees.

Nutritional analyses of the soils in the barren areas directly beneath the nests revealed that both total and plant‐available nutrients were the highest in these soils. Wheat plants from our phytometer experiment accumulated the most biomass when grown in these soils. This shows that the sociable weavers are providing an appropriate and available source of nutrients for plant growth, and yet other plants seem unable to establish in these soils in the wild. We observed the same barren areas below the nests on telephone poles (Figure 2); therefore, shading or faunal trampling (Greenwood & McKenzie, 2001; Lange, 1969; Natusch et al., 2017) is unlikely to be the major inhibitor of plant growth in this particular system. Instead, we suggest that the decreased infiltration rates of the soil below the nests prevent plants from establishing there, leading to the creation of these large barren patches in the subcanopy vegetation. The hydrophobicity of these soils could benefit to the deep‐rooted camel thorn trees in that competition from subcanopy vegetation is excluded. Furthermore, continuous deposition of feces by the weavers can lead to a change in soil microbial communities as has been previously shown to happen with penguin activity in maritime Antarctica (Guo et al., 2018). As such, changes in microbial communities can also potentially affect the establishment of plants (Hartman & Tringe, 2019; Raaijmakers, Paulitz, Steinberg, Alabouvette, & Moënne‐Loccoz, 2009) and lead to the observed barren areas directly beneath the nests (Guyonnet et al., 2017; Pochana & Keller, 1999).

While hosting a sociable weaver colony may alleviate some nutrient acquisition constraints and competition for camel thorn trees in this environment, we also found evidence of negative feedbacks. Key costs we documented were a 94% increase in summed cross‐sectional areas of fallen branches in trees that hosted weaver nests and a 14% reduction in foliar area of the tree canopy. Sociable weaver nests can become very large in size over time and extend onto multiple branches in camel thorn trees (Spottiswoode, 2009). In some instances, large sociable weaver colonies can completely replace the foliage of the branch on which they were built, and with increased nest mass (often after rain), these branches eventually break. Reductions in foliar area and increased broken branches suggest that hosting sociable weaver colonies do not come without severe long‐term costs for the trees. Future research will need to address how increasing colony size will impact the magnitude of costs to the trees. Additionally, since the nests are built mainly with dry plant material (from grasses such as *Aristida ciliata* and *A. obtusa*), the enormous amount of fuel in the event of a fire would increase the probability of tree mortality during such events (Mendelsohn & Anderson, 1997; Seymour & Huyser, 2008).

Our results suggest interesting potential costs and benefits of interactions between trees hosting fauna for any period of time. While the sociable weaver–camel thorn interaction likely presents an extreme case, nutrient inputs from other nesting fauna and potential use by the host trees or plants is likely a common feature in many systems. In arid, nutrient‐poor environments, this interaction may have significant implications for plant growth and reproduction, and potentially for associated vegetation (e.g., understorey plans). Ultimately, this may impact the spatial vegetation structure in the landscape. Given that plants providing structural diversity are often key ecosystem engineers in harsh arid environments due to their amelioration of conditions, the plant–animal interaction detailed in this study could have larger scale impacts and warrants further investigation.

## CONFLICT OF INTEREST

The authors have no competing interests.

## AUTHOR CONTRIBUTION

**Kervin Deveshwar Prayag:** Investigation (equal); Writing‐original draft (equal); Writing‐review & editing (equal). **Carla Jacqueline du Toit:** Investigation (equal); Writing‐original draft (equal); Writing‐review & editing (equal). **Michael Cramer:** Investigation (equal); Methodology (lead); Supervision (lead); Writing‐original draft (equal); Writing‐review & editing (equal). **Robert Thomson:** Conceptualization (lead); Funding acquisition (lead); Investigation (equal); Methodology (equal); Resources (lead); Supervision (lead); Writing‐original draft (equal); Writing‐review & editing (equal).

## Supporting information

Appendix S1

## Data Availability

The data analyzed and presented in this manuscript are available at: Prayag, Kervin; du Toit, Carla; Cramer, Michael; Thomson, Robert (2020), Faunal input at host plants: can Camel thorn trees use nutrients imported by resident Sociable weavers? Dryad, Dataset, https://doi.org/10.5061/dryad.1ns1rn8rt

## References

[ece36798-bib-0001] Anderson, W. B. , & Polis, G. (1999). Nutrient fuxes from water to land : Seabirds affect plant nutrient status on Gulf of California islands. Oecologia, 118(3), 324–332. 10.1007/s004420050733 28307276

[ece36798-bib-0002] Aranibar, J. N. , Macko, S. A. , Anderson, I. C. , Potgieter, A. L. F. , Sowry, R. , & Shugart, H. H. (2003). Nutrient cycling responses to fire frequency in the Kruger National Park (South Africa) as indicated by stable isotope analysis. Isotopes in Environmental and Health Studies, 39(2), 141–158. 10.1080/1025601031000096736 12872806

[ece36798-bib-0003] Archer, S. R. , Andersen, E. M. , Predick, K. I. , Schwinning, S. , Steidl, R. J. , & Woods, S. R. (2017). Woody plant encroachment: Causes and consequences In Briske D. (Ed.), Rangeland systems: Processes, management and challenges (pp. 25–84). Cham, Switzerland: Springer International Publishing 10.1007/978-3-319-46709-2

[ece36798-bib-0004] Barnes, R. D. , Fagg, C. W. , & Milton, S. J. (1997). Acacia erioloba: Monograph and annotated bibliography. Oxford, UK: Oxford Forestry Institute, University of Oxford.

[ece36798-bib-0005] Bates, D. , Mächler, M. , Bolker, B. , & Walker, S. (2015). Fitting linear mixed‐effects models using lme4. Journal of Statistical Software, 67(1), 1–48. 10.18637/jss.v067.i01

[ece36798-bib-0006] Belsky, A. J. (2002). Does herbivory benefit plants? A review of the evidence. The American Naturalist, 127(6), 870–892. 10.1086/284531

[ece36798-bib-0007] Bertness, M. D. , & Callaway, R. (1994). Positive interactions in communities. Trends in Ecology and Evolution, 9(5), 187–191. 10.1016/0169-5347(94)90087-6 21236818

[ece36798-bib-0008] Boeken, B. , Shachak, M. , Gutterman, Y. , & Brand, S. (1995). Patchiness and disturbance: Plant community responses to porcupine diggings in the central Negev. Ecography, 18(4), 410–421. 10.1111/j.1600-0587.1995.tb00144.x

[ece36798-bib-0009] Bronstein, J. L. (2015). Mutualisms. Oxford, UK: Oxford University Press.

[ece36798-bib-0010] Burke, A. (2006). Savanna trees in Namibia ‐ Factors controlling their distribution at the arid end of the spectrum. Flora ‐ Morphology, Distribution, Functional Ecology of Plants, 201(3), 189–201. 10.1016/j.flora.2005.06.011

[ece36798-bib-0011] Coates Palgrave, M. (2005). Keith Coates Palgrave Trees of Southern Africa, 3rd ed. Cape Town: Struik Publishers.

[ece36798-bib-0012] Cramer, M. D. , Chimphango, S. , De Fortier, A. , Waldram, M. , & Bond, W. (2007). Grass competition induces N_2_ fixation in some species of African Acacia. Journal of Ecology, 95(5), 1123–1133. 10.1111/j.1365-2745.2007.01285.x

[ece36798-bib-0013] Cramer, M. D. , & Midgley, J. J. (2015). The distribution and spatial patterning of mima‐like mounds in South Africa suggests genesis through vegetation induced aeolian sediment deposition. Journal of Arid Environments, 119, 16–26. 10.1016/j.jaridenv.2015.03.011

[ece36798-bib-0014] Cumming, G. , & Finch, S. (2005). Inference by eye confidence intervals and how to read pictures of data. American Psychologist, 60(2), 170–180. 10.1037/0003-066X.60.2.170 15740449

[ece36798-bib-0015] Davis, A. L. V. , Scholtz, C. H. , Kryger, U. , Deschodt, C. M. , & Strümpher, W. P. (2010). Dung beetle assemblage structure in Tswalu Kalahari Reserve: Responses to a mosaic of landscape types, vegetation communities, and dung types. Environmental Entomology, 39(3), 811–820. 10.1603/en09256 20550793

[ece36798-bib-0016] Dean, W. R. J. , Milton, S. J. , & Jeltsch, F. (1999). Large trees, fertile islands, and birds in arid savanna. Journal of Arid Environments, 41(1), 61–78. 10.1006/jare.1998.0455

[ece36798-bib-0017] Ellis, J. C. (2005). Marine birds on land: A review of plant biomass, species richness, and community composition in seabird colonies. Plant Ecology, 181(2), 227–241. 10.1007/s11258-005-7147-y

[ece36798-bib-0018] Erskine, P. D. , Bergstrom, D. M. , Schmidt, S. , Stewart, G. R. , Tweedie, C. E. , & Shaw, J. D. (1998). Subantarctic Macquarie Island: A model ecosystem for studying animal‐derived nitrogen sources using N‐15 natural abundance. Oecologia, 117(1–2), 187–193. 10.1007/s004420050647 28308485

[ece36798-bib-0019] Evans, R. D. (2001). Physiological mechanisms influencing plant nitrogen isotope composition. Trends in Plant Science, 6(3), 121–126. 10.1016/S1360-1385(01)01889-1 11239611

[ece36798-bib-0020] Ferraro, D. O. , & Oesterheld, M. (2002). Effect of defoliation on grass growth. A quantitative review. Oikos, 98, 125–133. 10.1034/j.1600-0706.2002.980113.x

[ece36798-bib-0021] Fox, J. , & Weisberg, S. (2019). An R Companion to applied regression. 3rd ed. Thousand Oaks CA: Sage Retrieved from https://socialsciences.mcmaster.ca/jfox/Books/Companion/

[ece36798-bib-0022] Garcia, L. V. , Maranon, T. , Ojeda, F. , Clemente, L. , & Redondo, R. (2002). Seagull influence on soil properties, chenopod shrub distribution, and leaf nutrient status in semi‐arid Mediterranean islands. Oikos, 98(1), 75–86. 10.1034/j.1600-0706.2002.980108.x

[ece36798-bib-0023] Gerlach, R. W. , Dobb, D. E. , Raab, G. A. , & Nocerino, J. M. (2002). Gy sampling theory in environmental studies. 1. Assessing soil splitting protocols. Journal of Chemometrics, 16(7), 321–328. 10.1002/cem.705

[ece36798-bib-0024] Greenwood, K. L. , & McKenzie, B. M. (2001). Grazing effects on soil physical properties and the consequences for pastures: A review. Australian Journal of Experimental Agriculture, 41, 1231–1250. 10.1071/ea00102

[ece36798-bib-0025] Grinath, J. B. , Larios, L. , Prugh, L. R. , Brashares, J. S. , & Suding, K. N. (2019). Environmental gradients determine the potential for ecosystem engineering effects. Oikos, 128(7), 994–1004. 10.1111/oik.05768

[ece36798-bib-0026] Guo, Y. , Wang, N. , Li, G. , Rosas, G. , Zang, J. , Ma, Y. , …, Cao, H. (2018). Direct and indirect effects of penguin feces on microbiomes in Antarctic ornithogenic soils. Frontiers in Microbiology, 9 10.3389/fmicb.2018.00552 PMC5891643 29666609

[ece36798-bib-0027] Guyonnet, J. P. , Vautrin, F. , Meiffren, G. , Labois, C. , Cantarel, A. A. M. , Michalet, S. , … Haichar, F. E. Z. (2017). The effects of plant nutritional strategy on soil microbial denitrification activity through rhizosphere primary metabolites. FEMS Microbiology Ecology, 93(4), 1–11. 10.1093/femsec/fix022 28334144

[ece36798-bib-0028] Harrell, F. E. , & Dupont, C. (2019). Hmisc: Harrell Miscellaneous. Retrieved from https://cran.r‐project.org/package=Hmisc

[ece36798-bib-0029] Hartman, K. , & Tringe, S. G. (2019). Interactions between plants and soil shaping the root microbiome under abiotic stress. Biochemical Journal, 476(19), 2705–2724. 10.1042/BCJ20180615 31654057 PMC6792034

[ece36798-bib-0030] Hernändez, L. M. A. , Sanders, J. G. , Miller, G. A. , Ravenscraft, A. , & Frederickson, M. E. (2017). Ant–plant mutualism: A dietary by‐product of a tropical ant’s macronutrient requirements. Ecology, 98(12), 3141–3151. 28977692 10.1002/ecy.2036

[ece36798-bib-0031] Hobara, S. , Osono, T. , Koba, K. , Tokuchi, N. , Fujiwara, S. , & Kameda, K. (2001). Forest floor quality and N transformations in a temperate forest affected by avian‐derived N deposition. Water, Air, and Soil Pollution, 130(1–4 II), 679–684. 10.1023/A:1013869115132

[ece36798-bib-0032] Kirshenbaum, I. , Smith, J. S. , Crowell, T. , Graff, J. , & McKee, R. (1947). Separation of the nitrogen isotopes by the exchange reaction between ammonia and solutions of ammonium nitrate. The Journal of Chemical Physics, 15(7), 440–446. 10.1063/1.1746562

[ece36798-bib-0033] Lambers, H. , Chapin, F. I. , & Pons, T. (2008). Plant Physiological Ecology. New York, NY: Springer‐Verlag New York.

[ece36798-bib-0034] Lange, R. T. (1969). The piosphere: Sheep track and dung patterns. Journal of Range Management, 22(6), 396–400. 10.2307/3895849

[ece36798-bib-0035] Lawrence, S. D. , Cooke, J. E. K. , Greenwood, J. S. , Korhnak, T. E. , & Davis, J. M. (2001). Vegetative storage protein expression during terminal bud formation in poplar. Canadian Journal of Forest Research, 31(6), 1098–1103. 10.1139/cjfr-31-6-1098

[ece36798-bib-0036] Lenth, R. V. (2019). emmeans: Estimated Marginal Means, aka Least‐Squares Means. Retrieved from https://cran.r‐project.org/package=emmeans

[ece36798-bib-0037] Lowney, A. M. (2020) Sociable weaver nests as a resource to local animal communities. PhD thesis. Cape Town, South Africa: Department of Biological Sciences, University of Cape Town.

[ece36798-bib-0038] Maclean, G. L. (1973). The sociable weaver, Part 2: Nest architecture and social organization. Ostrich, 44(3–4), 191–218. 10.1080/00306525.1973.9639159

[ece36798-bib-0039] Mendelsohn, J. M. , & Anderson, M. D. (1997). Sociable Weaver (*Philetairus socius*) In The Atlas of Southern African Birds, (Maclean), (pp. 534–535). Johannesburg: BirdLife South Africa.

[ece36798-bib-0040] Millard, P. (1988). The accumulation and storage of nitrogen by herbaceous plants. Plant, Cell & Environment, 11(1), 1–8. 10.1111/j.1365-3040.1988.tb01769.x

[ece36798-bib-0041] Mills, G. , & Mills, M. (2013). A natural guide to the Arid Kalahari including the Kgalagadi Transfrontier Park. Cape Town, South Africa: Africa Geographic Books.

[ece36798-bib-0042] Mizutani, H. , Hasegawa, H. , & Wada, E. (1986). High nitrogen isotope ratio for soils of seabird rookeries. Biogeochemistry, 2(3), 221–247. 10.1007/BF02180160

[ece36798-bib-0043] Mucina, L. , & Rutherford, M. C. (2006) . The vegetation of South Africa, Lesotho and Swaziland, Strelitzia 19 Pretoria, South Africa: South African National Biodiversity Institute.

[ece36798-bib-0044] Natusch, D. J. D. , Lyons, J. A. , Brown, G. , & Shine, R. (2016). Communally nesting migratory birds create ecological hot‐spots in Tropical Australia. PLoS One, 11(10), 1–13. 10.1371/journal.pone.0162651 PMC5051721 27706197

[ece36798-bib-0045] Natusch, D. J. D. , Mayer, M. , Lyons, J. A. , & Shine, R. (2017). Interspecific interactions between feral pigs and native birds reveal both positive and negative effects. Austral Ecology, 42(4), 479–485. 10.1111/aec.12465

[ece36798-bib-0046] O’Connor, T. G. , Puttick, J. R. , & Hoffman, M. T. (2014). Bush encroachment in southern Africa: Changes and causes. African Journal of Range and Forage Science, 31(2), 67–88. 10.2989/10220119.2014.939996

[ece36798-bib-0047] Osono, T. , Hobara, S. , Koba, K. , Kameda, K. , & Takeda, H. (2006). Immobilization of avian excreta‐derived nutrients and reduced lignin decomposition in needle and twig litter in a temperate coniferous forest. Soil Biology and Biochemistry, 38(3), 517–525. 10.1016/j.soilbio.2005.05.022

[ece36798-bib-0048] Pellmyr, O. (2002). Yuccas, Yucca Moths, and Coevolution: A Review. Annals of the Missouri Botanical Garden, 90(1), 35–55. 10.2307/3298524

[ece36798-bib-0049] Pinkalski, C. , Damgaard, C. , Jensen, K.‐M. , Peng, R. , & Offenberg, J. (2015). Quantification of ant manure deposition in a tropical agroecosystem: Implications for host plant nitrogen acquisition. Ecosystems, 18(8), 1373–1382. 10.1007/s10021-015-9906-5

[ece36798-bib-0050] Pochana, K. , & Keller, J. (1999). Study of factors affecting simultaneous nitrification and denitrification (SND). Water Science and Technology, 39(6), 61–68. 10.1016/S0273-1223(99)00123-7

[ece36798-bib-0051] R Core Team . (2020). R: A language and environment for statistical computing. Vienna, Austria: R Foundation for Statistical Computing http://www.r-project.org/index.html

[ece36798-bib-0052] Raaijmakers, J. M. , Paulitz, T. C. , Steinberg, C. , Alabouvette, C. , & Moënne‐Loccoz, Y. (2009). The rhizosphere: A playground and battlefield for soilborne pathogens and beneficial microorganisms. Plant and Soil, 321(1–2), 341–361. 10.1007/s11104-008-9568-6

[ece36798-bib-0053] Rohner, C. , & Ward, D. (1999). Large mammalian herbivores and the conservation of arid *Acacia* stands in the Middle East. Conservation Biology, 13(5), 1162–1171. 10.1046/j.1523-1739.1999.97300.x

[ece36798-bib-0054] Schlesinger, W. H. , Reynolds, J. F. , Cunningham, G. L. , Huenneke, L. F. , Jarrell, W. M. , & Virginia, R. A. (1990). Biological feedbacks in global desertification. Science, New Series, 247(4946), 1043–1048. 10.1126/science.247.4946.1043 17800060

[ece36798-bib-0055] Sekercioglu, Ç. H. , Wenny, D. G. , & Whelan, C. J. (2016). Why birds matter: Avian ecological function and ecosystem services, why birds matter. Chicago, IL: The University of Chicago Press 10.7208/chicago/9780226382777.001.0001

[ece36798-bib-0056] Seymour, C. L. (2008). Grass, rainfall and herbivores as determinants of *Acacia erioloba* (Meyer) recruitment in an African savanna. Plant Ecology, 197(1), 131–138. 10.1007/s11258-007-9366-x

[ece36798-bib-0057] Seymour, C. L. , & Huyser, O. (2008). Fire and the demography of camelthorn (*Acacia erioloba* Meyer) in the southern Kalahari ‐ Evidence for a bonfire effect? African Journal of Ecology, 46(4), 594–601. 10.1111/j.1365-2028.2007.00909.x

[ece36798-bib-0058] Shakesby, R. A. , Doerr, S. H. , & Walsh, R. P. D. (2000). The erosional impact of soil hydrophobicity: Current problems and future research directions. Journal of Hydrology, 231–232, 178–191. 10.1016/S0022-1694(00)00193-1

[ece36798-bib-0059] Smith, V. R. (1978). Animal‐plant‐soil nutrient relationships on Marion Island (Subantarctic). Oecologia, 32(2), 239–253. 10.1007/BF00366075 28309401

[ece36798-bib-0060] South African Weather Service . (2020). Retrieved from https://www.weathersa.co.za/ (Accessed: 28 June 2020).

[ece36798-bib-0061] Spottiswoode, C. N. (2009). Fine‐scale life‐history variation in sociable weavers in relation to colony size. Journal of Animal Ecology, 78(3), 504–512. 10.1111/j.1365-2656.2008.01507.x 19054222

[ece36798-bib-0062] Tilman, D. , Wedin, D. , & Knops, J. (1996). Productivity and sustainability influenced by biodiversity in grassland ecosystems. Nature, 379(6567), 718–720. 10.1038/379718a0

[ece36798-bib-0063] Tripler, C. , Canham, C. , Inouye, R. , & Schnurr, J. (2002). Soil nitrogen availability, plant luxury consumption, and herbivory by white‐tailed deer. Oecologia, 133(4), 517–524. 10.1007/s00442-002-1046-x 28466171

[ece36798-bib-0064] Venter, Z. S. , Cramer, M. D. , & Hawkins, H. J. (2018). Drivers of woody plant encroachment over Africa. Nature Communications, 9(1), 1–7. 10.1038/s41467-018-04616-8 PMC5995890 29891933

[ece36798-bib-0065] Whitney, K. D. (2002). Dispersal for distance? Acacia ligulata seeds and meat ants Iridomyrmex viridiaeneus. Austral Ecology, 27(6), 589–595. 10.1046/j.1442-9993.2002.01216.x

[ece36798-bib-0066] Wickham, H. (2016). ggplot2: Elegant graphics for data analysis. New York, NY: Springer‐Verlag.

[ece36798-bib-0067] Williams, S. J. , Arsenault, M. A. , Buczkowski, B. J. , Reid, J. A. , Flocks, J. , Kulp, M. A. , … Jenkins, C. J. (2006). Surficial Sediment Character of the Louisiana Offshore Continental Shelf Region: A GIS Compilation. Retrieved fromhttp://pubs.usgs.gov/of/2006/1195/index.htm.%0AAny

[ece36798-bib-0068] Young, V. A. (1936). Edaphic and vegetational changes associated with injury of a white pine plantation by roosting birds. Journal of Forestry, 34, 512–523.

[ece36798-bib-0069] Zółkoś, K. , Meissner, W. , Olszewski, T. S. , & Remisiewicz, M. (2013). Changes in Khasi pine (*Pinus kesiya* Royle ex Gordon) tree stands affected by Dimorphic Egret (*Egretta dimorpha*) colony at Madagascar. African Journal of Ecology, 51(2), 319–324. 10.1111/aje.12038

