# Supplementary material for: Faunal input at host plants: Can camel thorn trees use nutrients imported by resident sociable weavers?
Source: Ecol Evol. 2020 Sep 21;10(20):11643–56. doi: 10.1002/ece3.6798 (PMC7593193; doi:10.1002/ece3.6798)
Supplement: Supplementary file 1 — Appendix S1 [file ECE3-10-11643-s001.docx]

**List table and figures (including appendices)**

**Table 1** The mean δ^15^N values (‰) and nutrient concentrations (%) of sociable weaver faeces, with standard errors (n = 5).

**Figure 1 Left and top right panes:** sociable weaver colony nests on camelthorn trees at Tswalu Kahalari. **Right bottom pane:** sociable weaver coming out from nest chamber at Tswalu Kalahari. Photo credits: Anthony M. Lowney.

**Figure 2 Top left pane:** complete replacement of foliage on a branch of a camelthorn tree due to the presence of a sociable weaver nest; **Top right pane:** Branch breakage in a camelthorn tree as a direct consequence of hosting a sociable weaver nest; **Bottom left pane:** Barren area in the vegetation directly below a sociable weaver nest colony below a camelthorn tree; **Bottom right pane:** Barren area in the vegetation directly below a sociable weaver nest colony below a telephone pole.

**Figure 3** Variation in total N, P and K concentrations, and in δ^15^N in the top soil layer (0.1 m) in the barren areas directly below the nests, below control trees, in the periphery of both nest and control trees, in the grassland, and below nest and control telephone poles. The boxes and horizontal lines respectively represent the first and third quartiles, and the medians. The whisker represents 1.5 x the interquartile range and outliers above/below are shown as open circles. The diamonds represent the mean values. Different letters and indicate significant differences as determined by Tukey’s pair-wise comparisons.

**Figure 4** Soil concentrations of total N, P and K at depths of 0.1, 0.4 and 1.1 m below camelthorn trees (with and without sociable weaver nests) and in the open grassland, and below telephone poles, with and without nests. Points represent means, while coloured ribbons represent the bootstrapped 95% confidence intervals.

**Figure 5** The variation in soil δ^15^N values at depths of 0.1, 0.4 and 1.1 m, between soils under camelthorn trees (with and without sociable weaver nests), telephone poles (with and without nests), and in the open grassland. The vertical dashed line represents the mean δ^15^N value of the sociable weaver faecal samples (10.10 ‰ ± 0.35). Points represent means, while coloured ribbons represent the bootstrapped 95% confidence intervals.

**Figure 6** Soil concentrations of P (citric acid extraction), P-Olsen, K, pH and electrical conductivity in the top soil layer (0.1 m) below camelthorn trees with and without sociable weaver nests, in the grassland, and below telephone poles with and without nests . The boxes and horizontal lines represent the first and the third quartiles and the medians, respectively. The whisker represents 1.5 x the interquartile range and outliers above/below are shown as open circles. The diamonds represent the mean values. Different letters and indicate significant differences as determined by Tukey’s pair-wise comparisons.

**Figure 7** **a)** Infiltration rate in the top soil layer (0.1 m) below camelthorn trees with and without sociable weaver nests, and in the open grassland. The boxes and horizontal lines represent the first and the third quartiles and the medians, respectively. The whisker represents 1.5 x the interquartile range and outliers above/below are shown as open circles. The diamonds represent the mean values. Different letters and indicate significant differences as determined by Tukey’s pair-wise comparisons and **b)** mean cumulative change (since planting) in above-ground biomass (logged) of wheat grown in soils collected below camelthorn trees with and without sociable weaver nests, telephone poles with and without nests, and from the open grassland. Points represent means, while coloured ribbons represent the bootstrapped 95% confidence intervals.

**Figure 8** **a)** The variation in foliar δ^15^N between camelthorns with and without nests. The horizontal dashed line represents the mean δ^15^N value of the faecal samples, **b)** predicted foliar dry weight expressed as a function of the respective terminal branch lengths for camelthorn trees with and without sociable weaver nests as predicted by the fitted Linear Mixed-Effect Model, **c)** foliar dry weight expressed as a function of the respective terminal branch basal diameters as predicted by the fitted Linear Mixed-Effect Model, **d)** two-dimensional foliar areas expressed as a function of the cross-sectional areas of the respective ‘nest branches’ on camelthorn trees with and without nests **and e)** Cross-sectional area of fallen branches expressed as a function of cross-sectional areas at breast height of respective camelthorn trees with and without nests. The boxes and horizontal lines represent the first and the third quartiles and the medians, respectively. The whisker represents 1.5 x the interquartile range and outliers above/below are shown as open circles. The diamonds represent the mean values. Different letters and indicate significant differences as determined by Tukey’s pair-wise comparisons.

**Table S1** Results of the analyses of the top soil layer δ^15^N and elemental properties below nest trees, control trees and in the surrounding grassland. The presence of a ‘*****’ indicates a significant P value.

**Table S2** Results of the analyses of the top soil layer δ^15^N and elemental properties below nest and control telephone poles. The presence of a ‘*****’ indicates a significant P value. Singular fits are indicated by ‘n/a’ in the random effects column.

**Table S3** Results of the analyses of the soil elemental properties below nest trees, control trees and in the surrounding grassland at the different depths sampled. The presence of a ‘*****’ indicates significant P values.

**Table S4** Results of the analyses of the soil δ^15^N and elemental properties below nest and control telephone poles at the different depths sampled. The presence of a ‘*****’ indicates significant P values.

**Table S5** Results of the analyses of variation in P (citric acid extraction), P-Olsen, plant available K and Mg, pH and conductance in the top soil layer below nest trees, control trees and in the surrounding grassland. The presence of a ‘*****’ indicates a significant P value. Singular fits indicated by ‘n/a’ in the random effects column.

**Table S6** Results of the analyses of variation in P (citric acid extraction), P-Olsen, plant available K and Mg, pH and conductance in the top soil layer below nest and control telephone poles. The presence of a ‘*****’ indicates a significant P value. Singular fits indicated by ‘n/a’ in the random effects column.

**Table S7** Results of the analyses of the variation in soil δ^15^N and in the above-ground biomass (phytometer experiment) between nest and control trees, nest and control poles, and in the grassland, and of the variation in water infiltration rates in the soil below nest trees, control trees and in the grassland. The presence of a ‘*****’ indicates a significant P value.

**Table S8** Results of the analyses of the foliar nutrient concentrations between nest and control trees. The presence of a ‘*****’ indicates significant P values. Singular fits indicated by ‘n/a’ in random effects column.

**Table S9** Results of the analyses of the foliar physical properties between nest and control trees. The presence of a ‘*****’ indicates significant a P value.

**Figure S1** Variation in total concentrations of Ca, Cu, Zn, Mn and Fe in the top soil layer (0.1 m) in the barren areas directly below the nests, below control trees, in the periphery of both nest and control trees, in the grassland, and below nest and control telephone poles. The boxes and horizontal lines represent the first and the third quartiles and the medians, respectively. The whisker represents 1.5 x the interquartile range and outliers above/below are shown as open circles. The diamonds represent the mean values. Different letters and indicate significant differences as determined by Tukey’s pair-wise comparisons.

**Figure S2** Soil concentrations of Ca, Cu, Zn, Mn and Fe under camelthorn trees with and without sociable weaver nests, and in the grassland, at depths 0.1, 0.4 and 1.1 m. Points represent means, while coloured ribbons represent the bootstrapped 95% confidence intervals.

**Figure S3** Soil concentrations of Ca, Cu, Zn, Mn and Fe under telephone poles with and without nests, at depths 0.1, 0.4 and 1.1 m. Points represent means, while coloured ribbons represent the bootstrapped 95% confidence intervals.

**Figure S4** Soil texture of the top layers (0.1 m) of the soil by volume, for the nests in camelthorn trees and the nests on telephone poles. Coloured lines show mean values for each sample site, and grey areas indicate region of standard error.

**Table S10** Comparisons of the mean ± SE of texture components of the top soil (0.1 m) collected below the nest tree, control tree, nest pole, control pole and in the grassland. Different letters indicate significant differences as determined by Tukey’s pair-wise comparisons. Each texture class was analysed separately.

**Figure S5** Foliar concentrations of N, P, K, Mg, Ca, Cu, Zn, Mn and Fe in camelthorn trees with and without sociable weaver nests. The boxes and horizontal lines represent the first and the third quartiles and the medians, respectively. The whisker represents 1.5x the interquartile range and outliers above/below are shown as open circles. Different letters and indicate significant differences as determined by Tukey’s pair-wise comparisons**.**

**Table S1**

| **Response variable** | **Explanatory variable(s)** | **Random effect(s)** | **df** | **F value** | **P value** |
| --- | --- | --- | --- | --- | --- |
| **δ^15^N** | Location | Triplicate | 16 | 31.991 | < 0.001* |
|  |  |  |  |  |  |
| **N** | Location | Triplicate | 16 | 25.436 | < 0.001* |
|  |  |  |  |  |  |
| **P** | Location | Triplicate | 36 | 107.313 | < 0.001* |
|  |  |  |  |  |  |
| **K** | Location | Triplicate | 36 | 6.227 | 0.001* |
|  |  |  |  |  |  |
| **Ca** | Location | Triplicate | 36 | 10.829 | < 0.001* |
|  |  |  |  |  |  |
| **Cu** | Location | Triplicate | 36 | 8.643 | < 0.001* |
|  |  |  |  |  |  |
| **Zn** | Location | Triplicate | 36 | 27.724 | < 0.001* |
|  |  |  |  |  |  |
| **Mn** | Location | Triplicate | 36 | 8.878 | < 0.001* |
|  |  |  |  |  |  |
| **Fe** | Location | Triplicate | 36 | 0.880 | 0.485 |

**Table S2**

| **Response variable** | **Explanatory variable(s)** | **Random effect(s)** | **df** | **F value** | **P value** |
| --- | --- | --- | --- | --- | --- |
| **δ^15^N** | Location | Pair | 4 | 17.363 | 0.014 |
|  |  |  |  |  |  |
| **N** | Location | Pair | 4 | 27.665 | 0.006 |
|  |  |  |  |  |  |
| **P** | Location | Pair | 4 | 16.885 | 0.015 |
|  |  |  |  |  |  |
| **K** | Location | Pair | 4 | 6.504 | 0.063 |
|  |  |  |  |  |  |
| **Ca** | Location | n/a | 8 | 6.466 | 0.035 |
|  |  |  |  |  |  |
| **Cu** | Location | Pair | 4 | 13.606 | 0.021 |
|  |  |  |  |  |  |
| **Zn** | Location | Pair | 4 | 14.555 | 0.019 |
|  |  |  |  |  |  |
| **Mn** | Location | Pair | 4 | 0.084 | 0.786 |
|  |  |  |  |  |  |
| **Fe** | Location | Pair | 4 | 4.654 | 0.097 |

**Table S3**

| **Response variable** | **Explanatory variable(s)** | **Random effect(s)** | **df** | **F value** | **P value** |
| --- | --- | --- | --- | --- | --- |
| **N** | Location | Triplicate | 30 | 97.082 | < 0.001* |
|  | Depth |  | 30 | 124.095 | < 0.001* |
|  | Location*Depth |  | 30 | 9.060 | < 0.001* |
|  |  |  |  |  |  |
| **P** | Location | Triplicate | 72 | 99.912 | < 0.001* |
|  | Depth |  | 72 | 91.009 | < 0.001* |
|  | Location*Depth |  | 72 | 20.838 | < 0.001* |
|  |  |  |  |  |  |
| **K** | Location | Triplicate | 76 | 7.420 | < 0.001* |
|  | Depth |  | 76 | 0.090 | < 0.001* |
|  |  |  |  |  |  |
| **Ca** | Location | Triplicate | 72 | 5.498 | < 0.001* |
|  | Depth |  | 72 | 10.227 | < 0.001* |
|  | Location*Depth |  | 72 | 12.923 | < 0.001* |
|  |  |  |  |  |  |
| **Mn** | Location | Triplicate | 72 | 4.817 | < 0.001* |
|  | Depth |  | 72 | 8.411 | < 0.001* |
|  | Location*Depth |  | 72 | 2.807 | < 0.001* |
|  |  |  |  |  |  |
| **Fe** | Location | Triplicate | 76 | 1.168 | 3.17E-01 |
|  | Depth |  | 76 | 9.792 | < 0.001* |
|  |  |  |  |  |  |
| **Cu** | Location | Triplicate | 72 | 10.311 | < 0.001* |
|  | Depth |  | 72 | 5.583 | < 0.001* |
|  | Location*Depth |  | 72 | 3.908 | < 0.001* |
|  |  |  |  |  |  |
| **Zn** | Location | Triplicate | 72 | 33.876 | < 0.001* |
|  | Depth |  | 72 | 24.060 | < 0.001* |
|  | Location*Depth |  | 72 | 16.944 | < 0.001* |

**Table S4**

| **Response variable** | **Explanatory variable(s)** | **Random effect(s)** | **df** | **F value** | **P value** |
| --- | --- | --- | --- | --- | --- |
| **N** | Location | Pair | 16.969 | 19.061 | <0.001* |
|  | Depth |  | 18.499 | 12.131 | <0.001* |
|  |  |  |  |  |  |
| **P** | Location | Pair | 14.470 | 13.794 | 0.002* |
|  | Depth |  | 15.181 | 14.992 | <0.001* |
|  | Location*Depth |  | 14.470 | 5.903 | 0.013* |
|  |  |  |  |  |  |
| **K** | Location | Pair | 15.969 | 3.758 | 0.070* |
|  | Depth |  | 16.168 | 1.525 | 0.247 |
|  |  |  |  |  |  |
| **Ca** | Location | Pair | 14.532 | 1.453 | 0.247 |
|  | Depth |  | 15.884 | 2.122 | 0.153 |
|  | Location*Depth |  | 14.532 | 3.991 | 0.042* |
|  |  |  |  |  |  |
| **Mn** | Location | Pair | 15.934 | 2.835 | 0.112 |
|  | Depth |  | 16.377 | 3.076 | 0.073 |
|  |  |  |  |  |  |
| **Fe** | Location | Pair | 13.995 | 1.384 | 0.259 |
|  | Depth |  | 14.242 | 0.807 | 0.466 |
|  | Location*Depth |  | 13.995 | 1.548 | 0.247 |
|  |  |  |  |  |  |
| **Cu** | Location | Pair | 15.562 | 11.886 | 0.003* |
|  | Depth |  | 17.532 | 3.750 | 0.044* |
|  |  |  |  |  |  |
| **Zn** | Location | Pair | 14.431 | 3.546 | 0.080 |
|  | Depth |  | 15.785 | 13.664 | <0.001* |
|  | Location*Depth |  | 14.431 | 4.164 | 0.037* |

**Table S5**

| **Response variable** | **Explanatory variable(s)** | **Random effect(s)** | **df** | **F value** | **P value** |
| --- | --- | --- | --- | --- | --- |
| **P citric acid** | Location | n/a | 27 | 24.665 | < 0.001* |
|  |  |  |  |  |  |
| **P-Olsen** | Location | n/a | 27 | 43.335 | < 0.001* |
|  |  |  |  |  |  |
| **pH** | Location | n/a | 27 | 1.871 | 0.173 |
|  |  |  |  |  |  |
| **Conductance** | Location | n/a | 27 | 39.815 | < 0.001* |
|  |  |  |  |  |  |
| **K** | Location | n/a | 27 | 28.723 | < 0.001* |
|  |  |  |  |  |  |
| **Mg** | Location | n/a | 27 | 13.524 | < 0.001* |

**Table S6**

| **Response variable** | **Explanatory variable(s)** | **Random effect(s)** | **df** | **F value** | **P value** |
| --- | --- | --- | --- | --- | --- |
| **P citric acid** | Location | Pair | 4 | 60.742 | 0.001* |
|  |  |  |  |  |  |
| **P-Olsen** | Location | Pair | 4 | 36.761 | 0.004* |
|  |  |  |  |  |  |
| **pH** | Location | Pair | 4 | 0.260 | 0.637 |
|  |  |  |  |  |  |
| **Conductance** | Location | Pair | 4 | 128.281 | < 0.001* |
|  |  |  |  |  |  |
| **K** | Location | n/a | 8 | 7.665 | 0.024* |
|  |  |  |  |  |  |
| **Mg** | Location | n/a | 8 | 7.260 | 0.027* |

**Table S7**

| **Response variable** | **Explanatory variable(s)** | **Random effect(s)** | **df** | **F value** | **P value** |
| --- | --- | --- | --- | --- | --- |
| **Soil δ^15^N** | Location | Triplicate or pair | 28.716 | 47.754 | < 0.001* |
|  | Depth |  | 55.846 | 31.865 | < 0.001* |
|  |  |  |  |  |  |
|  |  |  |  |  |  |
| **Above-ground biomass** | Location | Triplicate or pair; Time | 45.688 | 44.780 | < 0.001* |
|  |  |  |  |  |  |
|  |  |  |  |  |  |
| **Infiltration rate** | Location | Triplicate | 48.000 | 23.203 | < 0.001* |

**Table S8**

| **Response variable** | **Explanatory variable(s)** | **Random effect(s)** | **df** | **F value** | **P value** |
| --- | --- | --- | --- | --- | --- |
| **Foliar δ^15^N** | Colony present (Yes/No) | Pair | n/a | 19.629 | < 0.001* |
|  |  |  |  |  |  |
| **N** | Colony Present (Yes/No) | Pair | 17 | 1.058 | 0.318 |
|  |  |  |  |  |  |
| **P** | Colony Present (Yes/No) | Pair | 17 | 0.219 | 0.646 |
|  |  |  |  |  |  |
| **K** | Colony Present (Yes/No) | Pair | 17 | 0.004 | 0.952 |
|  |  |  |  |  |  |
| **Ca** | Colony Present (Yes/No) | Pair | 17 | 3.445 | 0.081 |
|  |  |  |  |  |  |
| **Mn** | Colony Present (Yes/No) | Pair | 17 | 6.962 | 0.017* |
|  |  |  |  |  |  |
| **Fe** | Colony Present (Yes/No) | Pair | 17 | 1.437 | 0.247 |
|  |  |  |  |  |  |
| **Cu** | Colony Present (Yes/No) | n/a | 34 | 1.084 | 0.305 |
|  |  |  |  |  |  |
| **Zn** | Colony Present (Yes/No) | Pair | 17 | 1.435 | 0.247 |

**Table S9**

| **Response variable** | **Explanatory variable(s)** | **Random effect(s)** | **df** | **F value** | **P value** |
| --- | --- | --- | --- | --- | --- |
| **Foliar dryweight per unit length** | Colony present (Yes/No) | Tree within pair | 17 | 5.168 | 0.036* |
|  |  |  |  |  |  |
| **Foliar dryweight per unit diameter** | Colony present (Yes/No) | Tree within pair | 17 | 6.704 | 0.019* |
|  |  |  |  |  |  |
| **Foliar area on nest branch** | Colony present (Yes/No) | Pair | 17 | 5.447 | 0.032* |
|  |  |  |  |  |  |
| **Fallen branches** | Colony present (Yes/No) | Pair | 17 | 8.164 | 0.011* |
|  |  |  |  |  |  |
|  |  |  |  |  |  |


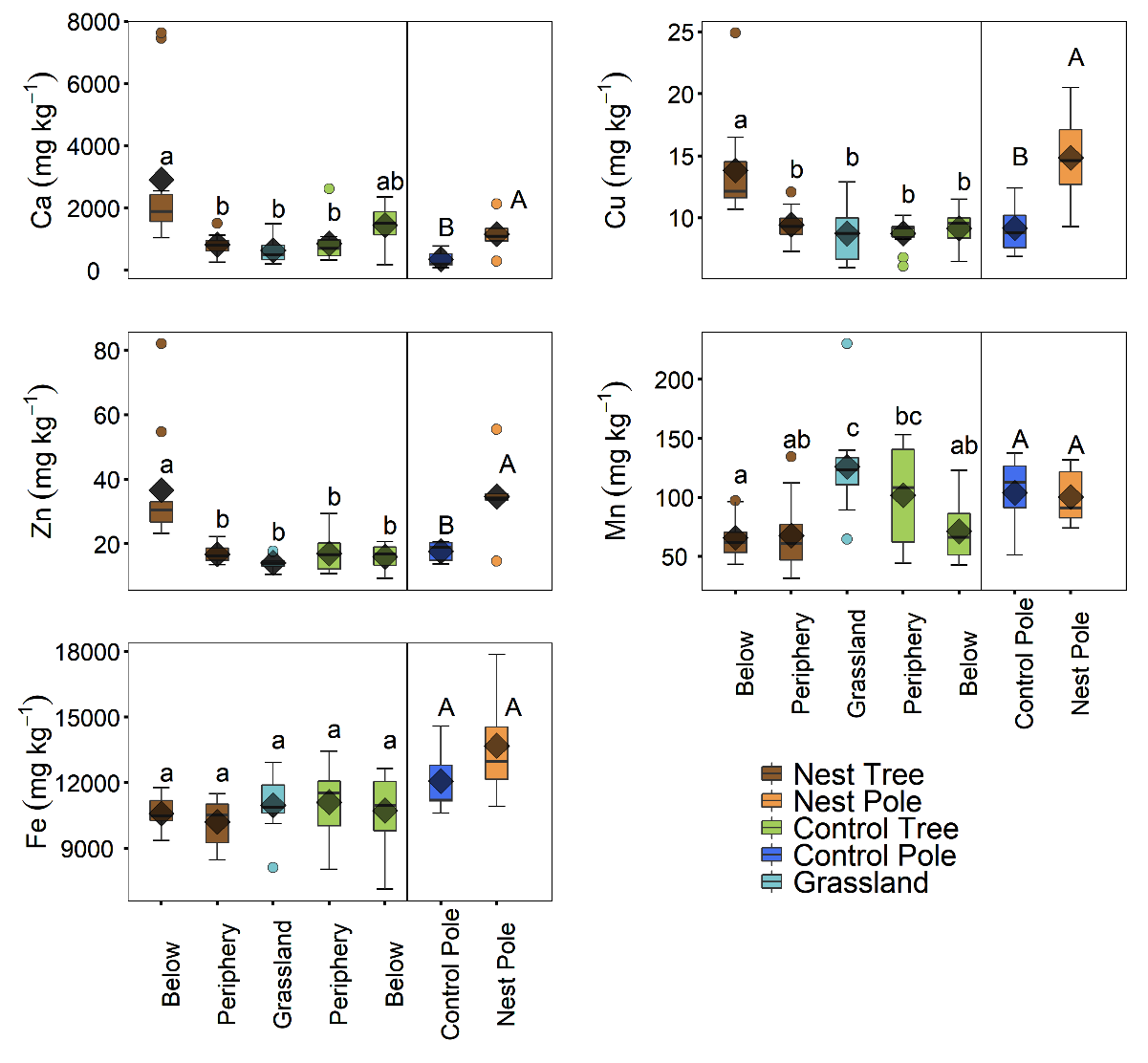


**Figure S1**


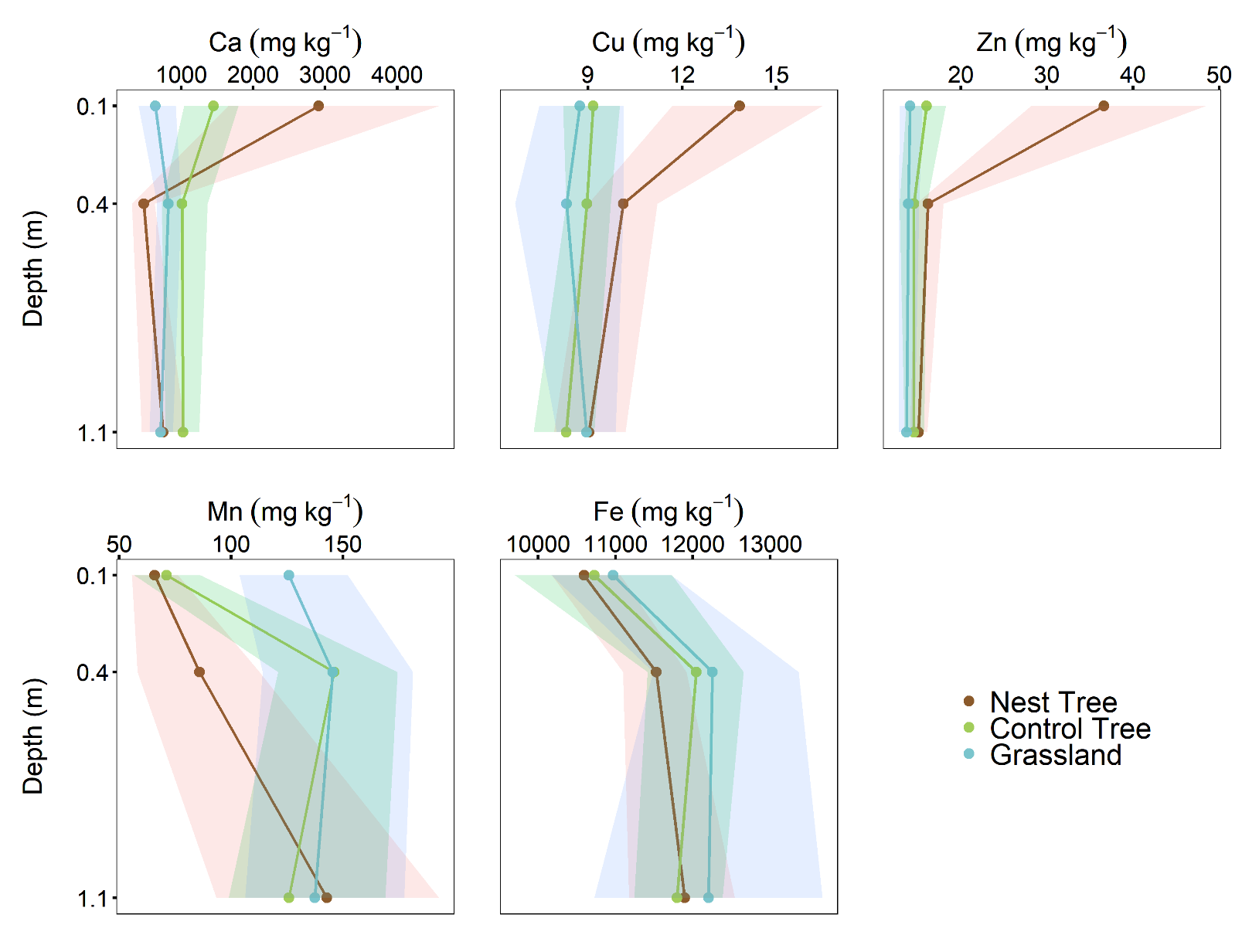


**Figure S2**

**
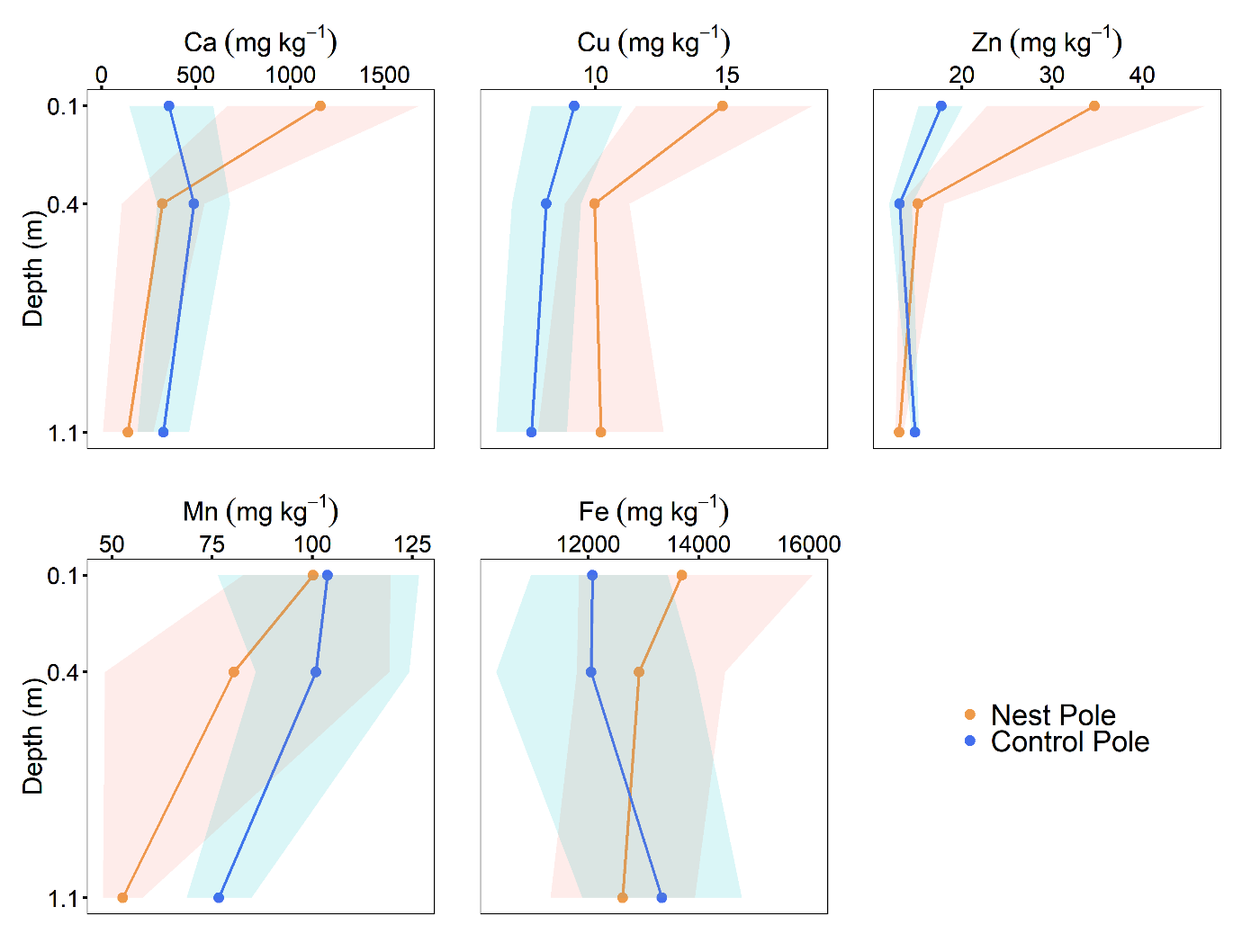
**

**Figure S3**

**
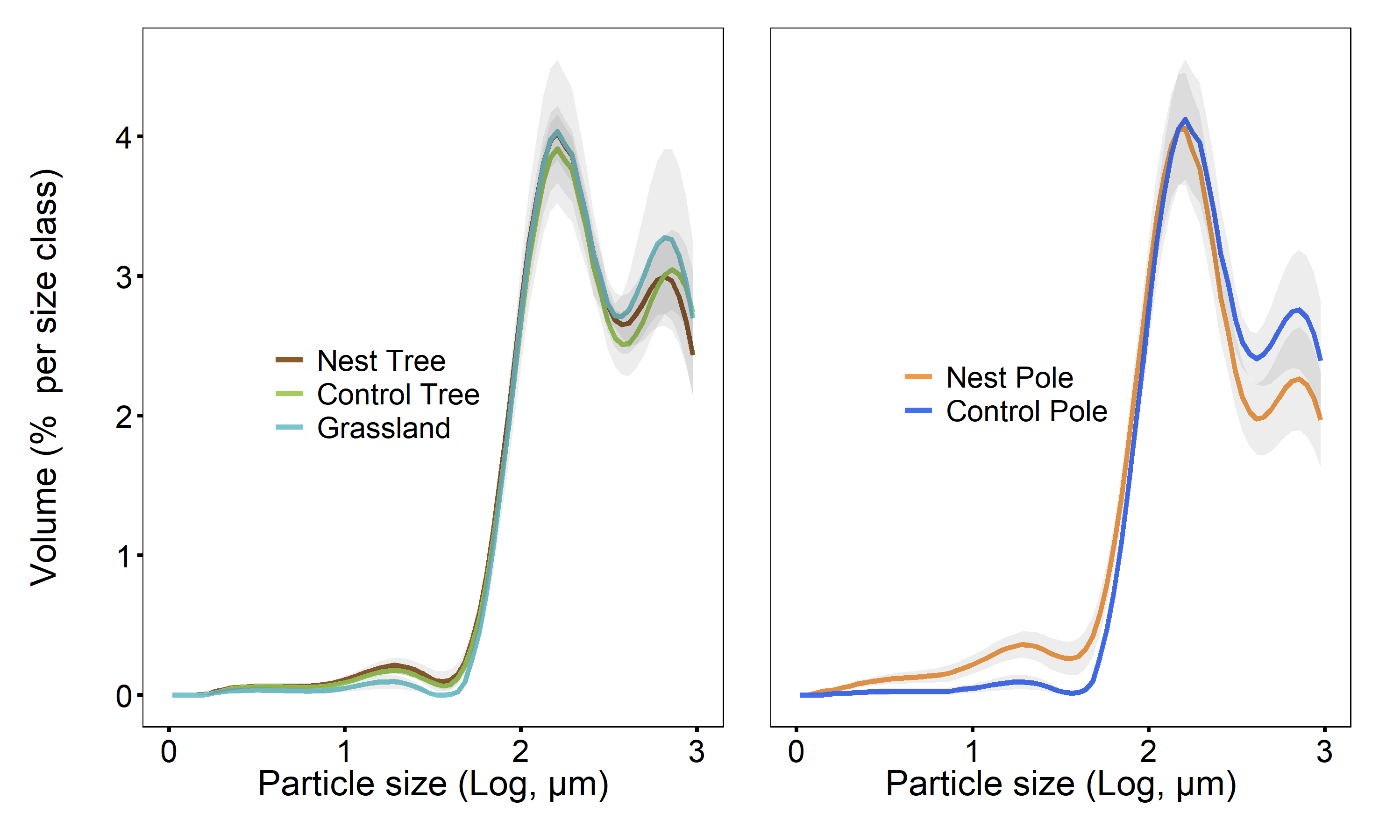
**

**Figure S4**

**Table S10**

|  | **Nest Tree** | **Control Tree** | **Grassland** | **Nest Pole** | **Control Pole** |
| --- | --- | --- | --- | --- | --- |
| **Clay** | 0.49±0.06_a_ | 0.44±0.05_ab_ | 0.29±0.08_b_ | 0.96±0.17_A_ | 0.25±0.11_B_ |
| **Silt** | 4.6±0.67_a_ | 3.8±0.27_a_ | 0.64±0.41_b_ | 8.5±1.4_A_ | 2.4±0.45_B_ |
| **Very fine sand** | 17.7±0.59_a_ | 16.9±0.60_a_ | 17.2±1.2_a_ | 20.5±0.97_A_ | 18.0±0.80_B_ |
| **Fine sand** | 29.9±0.73_a_ | 29.0±0.89_a_ | 30.0±1.8_a_ | 31.1±1.6_A_ | 31.5±1.7_A_ |
| **Medium sand** | 19.3±0.69_a_ | 18.4±0.72_a_ | 19.7±0.52_a_ | 15.8±0.87_A_ | 18.8±0.58_B_ |
| **Coarse sand** | 19.7±0.93_a_ | 20.5±0.95_a_ | 21.4±2.1_a_ | 15.6±1.3_A_ | 19.6±1.5_B_ |
| **Very coarse sand** | 8.3±0.82_a_ | 11.0±0.96_a_ | 9.2±0.92_a_ | 7.5±0.78_A_ | 9.4±1.03_B_ |


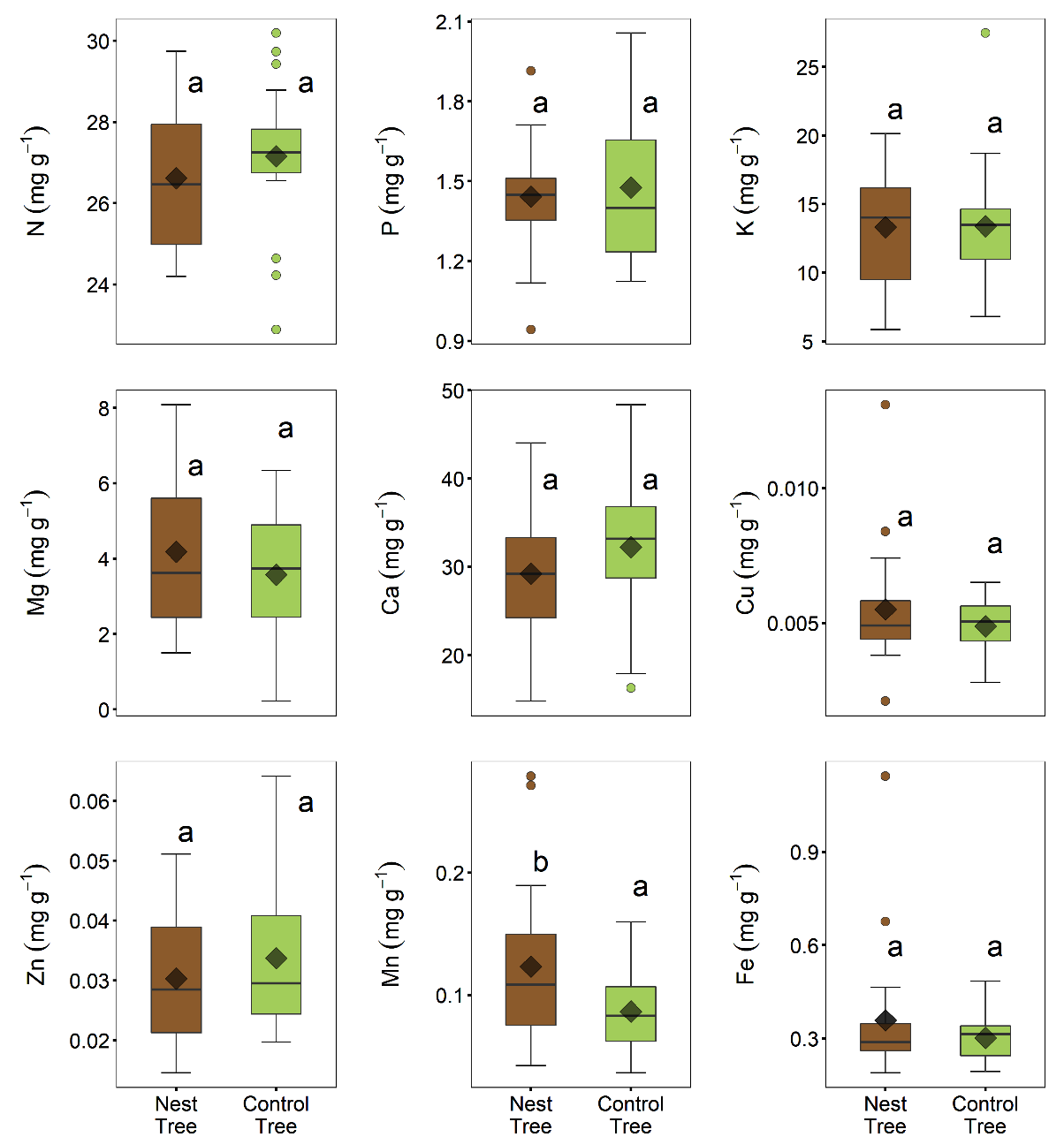


**Figure S5**
